# Next-generation morphological character discovery and evaluation: an X-ray micro-CT enhanced revision of the ant genus *Zasphinctus* Wheeler (Hymenoptera, Formicidae, Dorylinae) in the Afrotropics

**DOI:** 10.3897/zookeys.693.13012

**Published:** 2017-08-23

**Authors:** Francisco Hita Garcia, Georg Fischer, Cong Liu, Tracy L. Audisio, Evan P. Economo

**Affiliations:** 1 Okinawa Institute of Science and Technology Graduate University, 1919–1 Tancha, Onna-son 904–0495, Japan

**Keywords:** 3D model, cuticle, cybertype, micro-CT, morphology, mouthparts, new species, taxonomy

## Abstract

New technologies for imaging and analysis of morphological characters offer opportunities to enhance revisionary taxonomy and better integrate it with the rest of biology. In this study, we revise the Afrotropical fauna of the ant genus *Zasphinctus* Wheeler, and use high-resolution X-ray microtomography (micro-CT) to analyse a number of morphological characters of taxonomic and biological interest. We recognise and describe three new species: *Z. obamai* **sp. n.**, *Z. sarowiwai* **sp. n.**, and *Z. wilsoni* **sp. n.** The species delimitations are based on the morphological examination of all physical specimens in combination with 3D scans and volume reconstructions. Based on this approach, we present a new taxonomic discrimination system for the regional fauna that consists of a combination of easily observable morphological characters visible at magnifications of around 80–100 ×, less observable characters that require higher magnifications, as well as characters made visible through virtual dissections that would otherwise require destructive treatment. *Zasphinctus* are rarely collected ants and the material available to us is comparatively scarce. Consequently, we explore the use of micro-CT as a non-invasive tool for the virtual examination, manipulation, and dissection of such rare material. Furthermore, we delineate the treated species by providing a diagnostic character matrix illustrated by numerous images and supplement that with additional evidence in the form of stacked montage images, 3D PDFs and 3D rotation videos of scans of major body parts and full body (in total we provide 16 stacked montage photographs, 116 images of 3D reconstructions, 15 3D rotation videos, and 13 3D PDFs). In addition to the comparative morphology analyses used for species delimitations, we also apply micro-CT data to examine certain traits, such as mouthparts, cuticle thickness, and thoracic and abdominal muscles in order to assess their taxonomic usefulness or gain insights into the natural history of the genus. The complete datasets comprising the raw micro-CT data, 3D PDFs, 3D rotation videos, still images of 3D models, and coloured montage photos have been made available online as cybertypes (Dryad, http://dx.doi.org/10.5061/dryad.4s3v1).

## Introduction

The primary goal of taxonomic science is to organize life by developing hypotheses delimiting species and higher groups (Winston 1999; Wägele et al. 2011), but a secondary goal is to generate and curate information about species that can be useful for future taxonomic work as well as the broader fields of biology (Wheeler and Valdecasas 2007). The moment of description is particularly important, at which time an account of the species, supported and illustrated with information of some nature, is put on the record for posterity (Wheeler et al. 2004). The emergence of new technologies offers new opportunities for enhancing taxonomic descriptions and broadening their utility for other biological disciplines (La Salle et al. 2009; Schlick-Steiner et al. 2010; Faulwetter et al. 2013). Here, we continue a recent series of taxonomic works (Fischer et al. 2016; Sarnat et al. 2016; Hita Garcia et al. 2017a) exploring the use of three-dimensional (3D) data generated from X-Ray microcomputed tomography (micro-CT) for ant taxonomy, with a revision of the Afrotropical species of the genus *Zasphinctus* Wheeler. In our treatment of this group, we focus on exploring the potential for using micro-CT data for new character discovery and evaluation, enhancing the way descriptions themselves are organized and presented given the new data, and identifying anatomical characters that can be linked to the biology and ecology of the organism.

Micro-CT is a powerful imaging technology that enables the generation of high-resolution, virtual, and interactive 3D reconstructions of whole specimens or parts thereof. Such reconstructions can be virtually rotated, sectioned, measured, and dissected, thus allowing a comprehensive 3D analysis of the anatomy and morphology of the studied organisms (e.g. Faulwetter et al. 2013; Friedrich et al. 2014). Since its initial use for the study of insect cephalic morphology (Hörnschemeyer et al. 2002), micro-CT has gradually gained popularity as a fundamental tool for a variety of research fields that rely on the exact examination of animal morphology. It has been primarily employed for comparative and functional morphology (e.g. Beutel et al. 2008 2010; Zimmermann et al. 2011; Wojcieszek et al. 2012; Lipke et al. 2015), but also for the study of insect fossils in amber (Dierick et al. 2007; Barden and Grimaldi 2012), forensic entomology (Richards et al. 2012), and developmental biology (Metscher 2009). Surprisingly late, micro-CT has also been applied for invertebrate taxonomy of myriapods (Stoev et al. 2013; Akkari et al. 2015), spiders (Michalik and Ramírez 2013), earthworms (Fernández et al. 2014), and flatworms (Carbayo et al. 2016; Carbayo and Lenihan 2016). Despite its great potential for the taxonomy of extant insects, it has so far only been used in lepidopterans (Simonsen and Kitching 2014) and ants (Fischer et al. 2016; Sarnat et al. 2016; Hita Garcia et al. 2017a, b).

Compared to traditional methods like histology, the use of micro-CT provides the means for a quick and non-invasive generation of almost artefact-free morphological raw data for visualisation in 3D (Faulwetter et al. 2013; Friedrich et al. 2014). The non-destructive nature of the technique is of crucial importance for systematic research since it permits the scanning of very rare species and/or museum material, and it can be very well applied to type material. One drawback of modern collections-based systematics is that often important reference or type material is not available or accessible for examination, thus effectively slowing taxonomic progress (Smith and Blagoderov 2012; Wheeler et al. 2012; Faulwetter et al. 2013). One recent development with the potential to improve this situation is the establishment of virtual natural history collections that provide rapid access to anatomically correct and permanent digital reconstructions of type material. Based on the idea of Godfray (2007) to create virtual types, Faulwetter et al. (2013) introduced the concept of “cybertypes” and proposed a workflow to generate such virtual collections. Shortly afterwards Stoev et al. (2013) and Akkari et al. (2015) used micro-CT scanning for the description of new myriapod species and presented the first invertebrate cybertypes. Recently, Hita Garcia et al. (2017a, 2017b) critically explored the use of micro-CT for ant taxonomy and proposed the first ant cybertypes. Another great advantage is the application of micro-CT for virtual dissections and character identification, which has proven successful for a variety of invertebrates, such as myriapods (Blanke and Wesener 2014), Neuroptera (Zimmermann et al. 2011), and dragonflies (Simonsen and Kitching 2014).

Compared to other insect groups, ant taxonomy is thoroughly founded on the morphology of the very simplified worker caste. Despite that several authors also examine reproductive castes (e.g. Boudinot 2014; Yoshimura and Fisher 2014; Probst et al. 2015), this is often not possible due to the sheer absence of reproductives in collections and unknown associations between castes. Consequently, ant taxonomy predominantly focuses on phenotypical differences between workers of different species (Ward 2010). Due to the simplified female morphology of the worker caste, the vast majority of studies use very basic characters for species delimitations, mostly setation, surface sculpture, size, and shape differences of few body parts, especially eyes, mandibles, propodeal spines and the waist segments (e.g. Fernández 2004; Sarnat 2008; Bolton 2007; Fischer et al. 2012; Branstetter 2013; Hita Garcia et al. 2014). This approach offers the advantage that taxonomic studies of ants are relatively easy to perform. However, such a simplified approach increasingly often reaches its limits and cannot provide discriminatory evidence for species delimitations. Integrative taxonomy approaches including morphometrics (e.g. Csösz and Schulz 2010; Csösz and Fisher 2015), molecular phylogenetics (e.g. Branstetter 2012; Blaimer 2012), next generation sequencing (Fischer et al. 2015; Jesovnik et al. 2017), or combinations of multiple data sources (e.g. Schlick-Steiner et al. 2010; Csösz et al. 2014), have proven efficient to resolve the relationships within morphologically challenging ant genera. Nevertheless, in recent years, there have been very few approaches of advancing and improving traditional worker-based character sets used for species diagnostics (e.g. Bolton and Fisher 2012; Yoshimura and Fisher 2014).

The ant genus *Zasphinctus* Wheeler is a moderately small genus distributed in the Afrotropical, Indomalayan, and Australasian regions. Currently, 20 valid species are recognised (Bolton 2017), of which the vast majority occur in the Australasian region (15 spp. from Australia, one from New Caledonia, and one from New Guinea). By contrast, there is only one species known from South East Asia and two from the Afrotropical region. These ants are rarely collected and the material housed in natural history collections is somewhat limited. Perhaps due to its moderate species richness and relative rarity of collections, knowledge about the biology of *Zasphinctus* is rather incomplete. Wilson (1958) and Brown (1975) provided field notes about the biology of *Z. steinheili* (Forel) and laboratory observations of *Z. caledonicus* (Wilson). Both species turned out to be myrmecophagous feeding on larvae and adults of a variety of ant species captured during nest raids. Later, Buschinger (1989) confirmed this behaviour in *Z. steinheili* under laboratory conditions. Based on data from *Z. steinheili* and *Z. caledonicus*, colonies are found in soil and range in size from 100 to 500 workers. However, whether or not this is true for other *Zasphinctus* species, especially outside Australia, remains unknown.

A taxonomic problem commonly encountered in doryline ants is the existence of two or even three parallel taxonomic systems: a female-based one, which often splits into worker-based and queen-based, and a male-based one (e.g. Wilson 1964; Jaitrong and Yamane 2011; Borowiec 2016). Workers and reproductives are rarely collected together, and usually only one caste is available for taxonomic evaluation, which creates great difficulties for the association of workers with queen and/or males. This situation is especially problematic in, but not restricted to, the army ant genera *Aenictus* Shuckard and *Dorylus* Fabricius (Wilson 1964; Gotwald 1995). Prior to this study the taxonomy of *Zasphinctus* in the Afrotropical region was solely based on two male-based species described more than a hundred years ago from West African savannahs (Santschi 1915). Since then, no further taxonomic studies on *Zasphinctus* were published and the scarce male-based or worker-based material in collections has been tentatively assigned to one of these male-based species without evidence of any association. Recent collections in Kenya (Hita Garcia et al. 2009), Mozambique, and Uganda have yielded worker-based material without any males, thus not associable to any valid species name. Consequently, in order to use the genus for ant diversity inventories or conservation studies, it is imperative to create a taxonomic system founded on the worker caste.

In this study, we provide a taxonomic revision of the genus for the Afrotropical region on the basis of the worker caste. All three species treated herein are newly described. The taxonomic decision-making was founded on the examination of all physical specimens, as well as on 3D volume reconstructions of high-resolution micro-CT scanning data from several specimens per species, if available. Based on that approach, our newly developed taxonomic discrimination system consists of a new character set, which is unusual in ant taxonomy. The backbone of it is still based on easily observable morphological characters visible at magnifications of around 80 to 100 ×. On the basis of micro-CT scanning data, we also present less perceivable characters that require higher magnifications, previously only achieved through scanning electron microscopy (SEM), as well as characters that are usually hidden or partly obscured and would require destructive treatment of the physical material. Through virtual dissections of 3D reconstructed specimens, we recovered several of these hidden characters. Furthermore, we present our results in a different way compared to previous ant taxonomy revisions by including numerous stacked montage images, micro-CT microphotographs, 3D PDFs, and 3D rotation videos of relevant body parts in addition to full specimens. We argue that such a wealth of illustrative power obviates the need for lengthy descriptions and a traditional identification key. Instead, we opt for a thorough genus description and brief species accounts supplemented by a detailed diagnostic species character matrix with high quality illustrations for all characters. Finally, we use micro-CT data to examine traits, such as mouthparts, cuticle thickness, thoracic and abdominal muscles, and the sting in order to gain insights into the natural history of the genus. The complete datasets comprising the micro-CT raw data, 3D PDFs, 3D rotation videos, and coloured montage photos have been made available online as cybertypes (Hita Garcia et al. 2017c).

## Material and methods

### Abbreviations of depositories

Institutional museum collection abbreviations follow Evenhuis (2017). The material on which this study is based is located and/or was examined at the following institutions:

**BMNH** The Natural History Museum, London, U.K.

**CASC** California Academy of Sciences, San Francisco, U.S.A.

**MCZC** Museum of Comparative Zoology, Harvard University, Cambridge, U.S.A.

**NMKE** National Museums of Kenya, Nairobi, Kenya

**ZFMK** Zoological Research Museum Alexander Koenig, Bonn, Germany

### Material examined and terminology

The general terminology for ant morphology predominantly follows Keller (2011) and to a lesser extent Bolton (1990) and Borowiec (2009, 2016). For the description of mouthparts, we used the terminology of Gotwald (1969) and Keller (2011). The terminology for the description of surface sculpturing follows Harris (1979).

### Montage images and line drawings

All raw images were taken with a Leica DFC450 camera attached to a Leica M205C microscope and Leica Application Suite (version 4.1). The raw photo stacks were then processed to single montage images with Helicon Focus (version 6). All montage images used in this publication are available online and can be seen on AntWeb. Vector illustrations were created with Adobe Illustrator (version CS 5) by tracing specimen photographs.

### Measurements and indices

**Figure 1.**
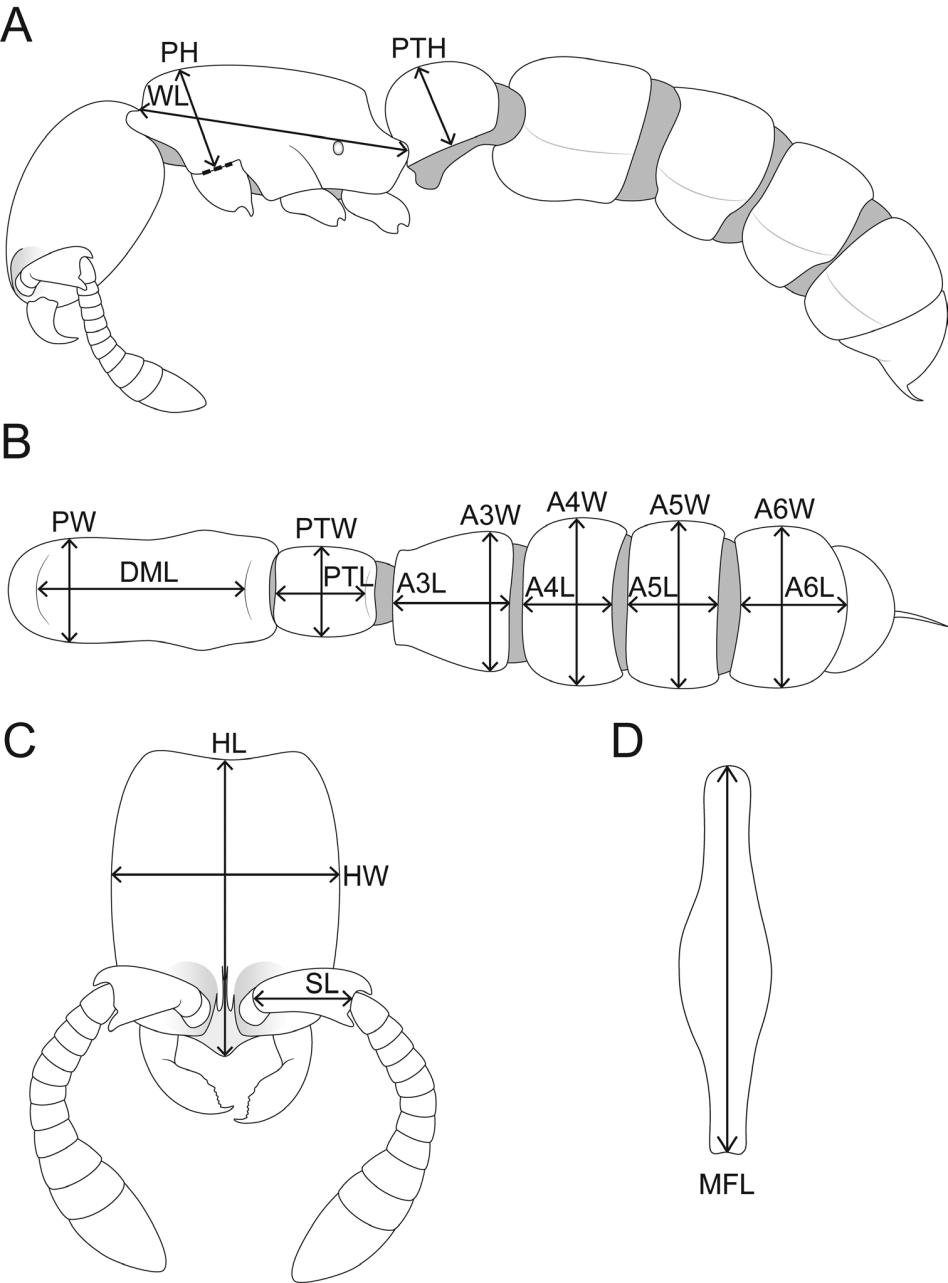
Schematic line drawings illustrating the measurements used in this study. **A** Body in profile with measuring lines for PH, PTH, and WL **B** Mesosoma and metasoma in dorsal view with measuring lines for A3L, A3W, A4L, A4W, A5L, A5W, A6L, A6W, DML, PW, PTL, and PTW **C** Head in full-face view with measuring lines for HL, HW, and SL **D** Metafemur in dorsal view with measuring line for MFL.

We measured 17 physical workers with a Leica M125 equipped with an orthogonal pair of micrometers under magnifications of 80 to 100 ×. Measurements and indices are presented as minimum and maximum values with arithmetic means in parentheses. In addition, measurements are expressed in mm to two decimal places. Since the workers of all three species treated herein are eyeless we omit any eye measurements and do not generate an ocular (or eye) index. We refrain from using total length since it is difficult to measure in already dry-mounted specimens that are not orientated in a straight line. The standard measurements HW and WL provide sufficient information about general body size dimensions. The following measurements and indices partly follow Bolton and Fisher (2012), Hita Garcia and Fischer (2014) and Hita Garcia et al. (2014) or are used here for the first time (Fig. 1):

**HL** Head Length: maximum distance from the midpoint of the anterior clypeal margin or from a line spanning the anterior-most points of the frontal lobes (depending on which projects farthest forward) to the midpoint of the posterior margin of head, measured in full-face view (Fig. 1C).

**HW** Head Width: the maximum width of the head capsule, measured in full-face view (Fig. 1C).

**SL** Scape Length: the maximum straight-line length of the scape, excluding the basal constriction or the neck (Fig. 1C).

**PH** Pronotal Height: the maximum height of the pronotum in profile (Fig. 1A).

**PW** Pronotal Width: the maximum width of the pronotum in dorsal view (Fig. 1B).

**DML** Dorsal Mesosoma Length: maximum length of mesosomal dorsum from anterodorsal margin of pronotum to dorsal margin of propodeal declivity (Fig. 1B).

**WL** Weber’s Length of Mesosoma: the maximum diagonal length of the mesosoma in profile, from the angle at which the pronotum meets the cervix to the posterior basal angle of the metapleuron (Fig. 1A).

**MFL** Metafemur Length: the maximum straight-line length of the metafemur, measured in dorsal view (Fig. 1D).

**PTL** Abdominal Segment II (petiole) Length: the maximum length of abdominal segment II (petiole), measured in dorsal view (Fig. 1B).

**PTH** Abdominal Segment II (petiole) Height: the maximum height of the petiolar tergum in profile view, including laterotergite, excluding petiolar sternum (Fig. 1A).

**PTW** Abdominal Segment II (petiole) Width: the maximum width of abdominal segment II (petiole), measured in dorsal view (Fig. 1B).

**A3L** Abdominal Segment III Length: the maximum length of abdominal segment III, measured in dorsal view (Fig. 1B).

**A3W** Abdominal Segment III Width: the maximum width of abdominal segment III, measured in dorsal view (Fig. 1B).

**A4L** Abdominal Segment IV Length: the maximum length of abdominal segment IV, measured in dorsal view (Fig. 1B).

**A4W** Abdominal Segment IV Width: the maximum width of abdominal segment IV, measured in dorsal view (Fig. 1B).

**A5L** Abdominal Segment V Length: the maximum length of abdominal segment V, measured in dorsal view (Fig. 1B).

**A5W** Abdominal Segment V Width: the maximum width of abdominal segment V, measured in dorsal view (Fig. 1B).

**A6L** Abdominal Segment VI Length: the maximum length of abdominal segment VI, measured in dorsal view (Fig. 1B).

**A6W** Abdominal Segment VI Width: the maximum width of abdominal segment VI, measured in dorsal view (Fig. 1B).

**CI** Cephalic Index: HW / HL × 100

**SI** Scape Index: SL / HL × 100

**DMI** Dorsal Mesosoma Index: PW / WL × 100

**DMI2** Dorsal Mesosoma Index 2: DML / WL × 100

**LMI** Lateral Mesosoma Index: PH / WL × 100

**MF** Metafemur Index: MFL / HW × 100

**LPI** Lateral Petiole Index: PTL / PTH × 100

**DPI** Dorsal Petiole Index: PTW / PTL × 100

**DA3I** Dorsal Abdominal Segment III Index: A3W / A3L × 100

**DA4I** Dorsal Abdominal Segment IV Index: A4W / A4L × 100

**DA5I** Dorsal Abdominal Segment V Index: A5W / A5L × 100

**DA6I** Dorsal Abdominal Segment VI Index: A6W / A6L × 100

### Micro X-ray computed tomography

Micro-CT scans were performed using a ZEISS Xradia 510 Versa 3D X-ray microscope and the ZEISS Scout and Scan Control System software (version 10.7.2936). The scanned specimens were left attached to their paper point, which was clamped to a holding stage. Scan settings were selected according to yield optimum scan quality: optical magnification of 4 ×, exposure times of 1–3 s, binning of two by two pixels, source filter “air”, voltage of 35–85 keV, power of 3–7.5 W, current of 71–88 µA, and field mode “normal”. The combination of voltage, power and exposure time was set to yield intensity levels of between 10,000 and 15,000 across the whole specimen. Scanning duration varied from 1.2 to 2.2 h, depending on exposure time. Full 360 degree rotations were done with a number of 1601 projections. The resulting scans have resolutions of 1013 × 1013 pixels and voxel sizes are in range of 0.94–4.6 µm. The original file size was 3113.577 MB for all scans. We scanned a varying number of specimens per species, depending on specimen availability and character suitability. An overview of the specimens used and scanning settings is provided in Table 1.

**Table 1.** Data summary for micro-CT scanning giving an overview of the specimens and body parts scanned for the three species and presenting specimen data, scan settings, and voxel sizes for the resulting scans (all specimens are workers and all files are in DICOM format).

Species	Body part scanned	Specimen identifier	voxel size (µm)	exposure time (s)	Power (W)	Voltage (kV)	Amperage (µA)
*Z. obamai*	full body	CASENT0764125	3.003	2	3	40	75
*Z. obamai*	head	CASENT0764127	0.945	3	6	70	85
*Z. obamai*	mesosoma	CASENT0764127	1.604	2	5	55	82
*Z. obamai*	metasoma	CASENT0764127	1.952	2	4	50	80
*Z. sarowiwai*	full body	CASENT0764650	4.606	1.8	4	50	81
*Z. sarowiwai*	mouthparts	CASENT0764652	0.945	3	6	65	84
*Z. sarowiwai*	full body	CASENT0764654	3.861	2	3	40	76
*Z. sarowiwai*	head	CASENT0764654	1.267	3	5	60	83
*Z. sarowiwai*	mesosoma	CASENT0764654	1.931	2	4	50	80
*Z. sarowiwai*	metasoma	CASENT0764654	2.834	1	4	45	78
*Z. wilsoni*	full body	MCZ-ENT-00512764	3.137	2.5	3	35	71
*Z. wilsoni*	head	MCZ-ENT-00512764	0.965	3	6	70	86
*Z. wilsoni*	mesosoma	MCZ-ENT-00512764	1.292	2.7	5	55	82
*Z. wilsoni*	metasoma	MCZ-ENT-00512764	2.312	2	4	45	78

### Virtual reconstruction and post-processing of raw data

3D reconstructions of the resulting scans were done with XMReconstructor (version 10.7.2936) and saved in DICOM file format (default settings; USHORT 16 bit output data type). Post-processing of DICOM raw data was performed with Amira software (version 6.1.1). Virtual examinations of 3D surface models were performed by using either the “volren” or “volume rendering” functions. The desired volume renderings were generated by adjusting colour space range to a minimum so that the exterior surface of specimens remained visible at the highest available quality. The 3D models were rotated and manipulated to allow a complete virtual examination of the scanned specimens. Images of shaded surface display volume renderings were made with the “snapshot” function at the highest achievable resolution (usually at around 1500 by 893 pixels). Volumetric surface rendering rotational videos of head, mesosoma, metasoma, and full body scans were created with the “camera path” object (5–10 keyframes, constant velocity for constant rotation speed) and “movie maker” function (parameters: MPEG format, AntiAlias2, total of 1200 frames at 60 frames per second, and resolution of 1920 × 1080 pixels).

### Character recognition and virtual dissections

In addition to the traditional morphological examination of the physical specimens under a light microscope with magnifications up to 100 ×, we virtually examined the full external morphology of the treated species in Amira. For this we compared more than 50 morphological characters potentially significant for dorylines (Bolton 1990; Keller 2011; Borowiec 2016), especially the genera previously grouped as Cerapachyinae, in the 3D models and made more than 350 snapshots to assess which characters have diagnostic value. A series of characters were hidden or obscured by other body parts, thus not observable by light microscopy, or only after destructive dissection. Due to the severe lack of material the latter was not an option. In order to examine such characters and explore a wider range of morphology, we used the segmentation function in Amira to deselect body parts obscuring segmented body parts. By doing so we were able to expose every desired structure. We examined the head capsule from all sides, which is usually ventrally and posteriorly obscured by antennae, legs, or anterior mesosoma, as well as ventral metasomal characters usually hidden between propodeum, legs, and different abdominal segments. Based on more than 110 character images per species, we chose to highlight 24 characters of high diagnostic significance for our newly developed species delimitation system (see Table 2 for complete list of examined characters). Furthermore, the volume reconstructions of the mouthparts and musculature were generated by using the segmentation function in Amira. Targeted structures were first visualised by adjusting density and contrast and then segmented by manually tracing their outline slide by slide.

**Table 2.** List of all important characters examined in this study with assessment of diagnostic potential and information on usage in this study (characters marked with * were used for species delimitations).

Characters examined	Diagnostic assessment and usage
**Head characters**	
Shape of head in full-face view	none, no significant interspecific variation observed, not used in this study
Shape of head in profile *	high, used in this study
Shape of mandibles	none, no significant interspecific variation observed, not used in this study
Mandibular dentition	none, no significant interspecific variation observed, not used in this study
Shape of clypeus	low, no significant interspecific variation observed, not used in this study
Presence of median clypeal tooth *	high, used in this study
Cuticular apron of clypeus	none, no significant interspecific variation observed, not used in this study
Torulo-posttorular complex *	high, used in this study
Antennal bulbus	none, no significant interspecific variation observed, not used in this study
Antennal scapes *	high, used in this study
Antennal pedicel and funiculus	none, no significant interspecific variation observed, not used in this study
Anterior tentorial pits	none, no significant interspecific variation observed, not used in this study
Parafrontal ridges *	high, used in this study
Eyes	none, absent in the worker caste
Vertex *	high, used in this study
Occipital margin in posterodorsal view *	high, used in this study
Occiput in posterior view *	high, used in this study
Occipital margin in posteroventral view *	high, used in this study
Hypostoma *	high, used in this study
Mouthparts (maxillae, labium, labrum)	unclear, none in closed in condition; described in open condition for *Z. sarowiwai*, but needs further investigation with better preserved alcohol material for µCT scanning
Tentorium (internal)	unclear, tentatively examined in this study and appears species-specific, but needs further investigation with better preserved alcohol material for µCT scanning
**Mesosoma characters**	
Mesosoma in profile *	high, used in this study
Endosternum (internal)	unclear, tentatively examined in this study and appears species-specific, but needs further investigation with better preserved alcohol material for µCT scanning
Transverse mesopleural groove	moderately variable among species, not used in this study
Propleuron	none, no significant interspecific variation observed, not used in this study
Pleural endophragmal pit *	high, used in this study
Mesopleuron	moderately variable among species, not used in this study
Metapleuron	low, no significant interspecific variation observed, not used in this study
Mesosoma dorsal *	high, used in this study
Probasitarsus	low, no significant interspecific variation observed, not used in this study
Calcar of strigil	low, no significant interspecific variation observed, not used in this study
**Metasoma characters**	
Levator of petiole	unclear, not examined in this study, very difficult to virtually dissect
Petiolar tergum in profile *	high, used in this study
Laterotergites	low, no significant interspecific variation observed, not used in this study
Subpetiolar process of petiolar sternum in profile *	high, used in this study
Petiolar tergum in dorsal view *	high, used in this study
Disc of petiole	none, no significant interspecific variation observed, not used in this study
Subpetiolar process in ventral view *	high, used in this study
Helcium	unclear, not examined in this study
Abdominal segment III in dorsal view *	high, used in this study
Abdominal segment III in ventral view *	high, used in this study
Posterior end of abdominal segment III in ventral view *	high, used in this study
Prora in anteroventral view *	high, used in this study
Abdominal segment IV in dorsal view	moderate, relatively variable within species, not used in this study
Abdominal segment IV in ventral view	moderate, relatively variable within species, not used in this study
Abdominal segment V in dorsal view	low, no significant interspecific variation observed, not used in this study
Abdominal segment V in ventral view	low, no significant interspecific variation observed, not used in this study
Abdominal segment VI in dorsal view *	high, used in this study
Abdominal segment VI in ventral view	high, not used in this study
Girdling constrictions abdominal segments IV, V, VI *	high, used in this study
Pygidium	low, no significant interspecific variation observed, not used in this study
Hypopygium	low, no significant interspecific variation observed, not used in this study
Spiracles abdominal segments II-VII	none, no significant interspecific variation observed, not used in this study
General surface sculpture *	high, used in this study
Cuticle thickness (internal)	unclear, examined in this study but needs further investigation with more specimens

### Virtual measuring of cuticle thickness

**Figure 2.**
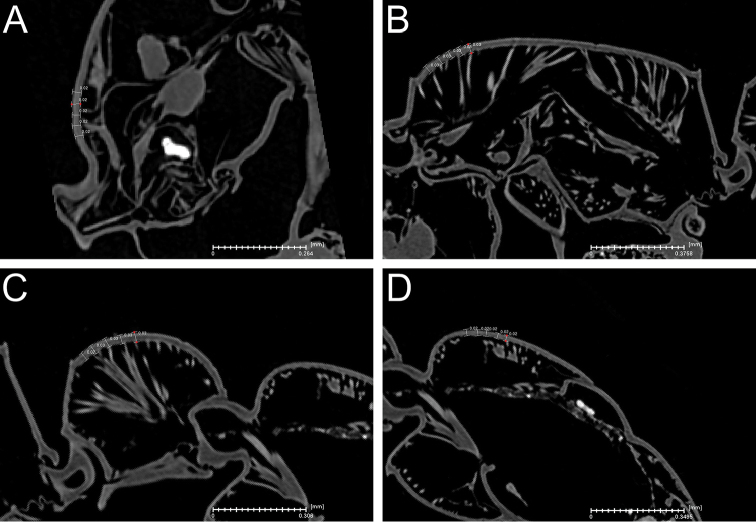
Microtomographic slides showing cuticle thickness measurements (with measuring lines in white). **A** Head in profile **B** Mesosoma in profile **C** Petiole (abdominal segment II) in profile **D** Abdominal segment III in profile.

In addition to the taxonomic standard measurements of external morphology given above, we also measured the thickness of the exoskeleton cuticle of the cephalic capsule, the pronotum, and abdominal segments II (petiole) and III. Measuring was performed with Amira by using the 2D measuring tool on slices representing sagittal sections along the median axis of the chosen body parts. For each body part, we measured five times over a defined area (Fig. 2) and calculated the average thickness. Based on Peeters et al. (2017) we put the measurements in context to body size by using the following measurements and indices:

Cephalic capsule cuticle thickness (CCC): thickness of cuticle of head measured in profile a short distance posterior of torulo-posttorular complex (Fig. 2A).

Dorsal pronotum cuticle thickness (PRC): thickness of cuticle of pronotum measured in profile a short distance posterior of anterodorsal margin (Fig. 2B).

Dorsal abdominal segment II (petiole) cuticle thickness (ASIIC): thickness of cuticle of abdominal segment II measured in profile a short distance posterior of anterodorsal margin (Fig. 2C).

Dorsal abdominal segment III cuticle thickness (ASIIIC): thickness of cuticle of abdominal segment III measured in profile a short distance posterior of anterodorsal margin (Fig. 2D).

Cephalic capsule cuticle thickness index (CCCI): CCC / HW × 1000

Dorsal pronotum cuticle thickness index (PRCI): PRC / HW × 1000

Dorsal abdominal segment II (petiole) cuticle thickness index (ASIICI): ASIIC / HW × 1000

Dorsal abdominal segment III cuticle thickness index (ASIIICI): ASIIIC / HW × 1000

### 3D PDFs

The first step to creating unicoloured 3D PDFs was to make 3D renderings of ant specimens in Amira using the Isosurface function (deselect compactify) for exporting surface meshes in the STL file format. These were imported into Meshlab (version 1.3.3) where the number of vertices per specimen was reduced in three steps to decrease total file size and before importing into Adobe Acrobat. First, the scan files were cleaned from isolated vertices (Filters > Cleaning and Repairing > Remove isolated pieces (wrt diameter) [set max diameter: 0.05–1%]) and the paper tips on which the ants are mounted were digitally removed as much as possible. The next step removed all internal vertices so that only the exoskeleton remained (1. Filters > Color Creation and Processing > Ambient Occlusion Per Vertex; 2. Filters > Selection > Select Faces By Vertex Quality (min = 0, max = 0.001); 3. Remove Selected Faces). In the last step, the number of total vertices was reduced to the final number of <750,000 (Filters > Remeshing, Simplification and Reconstruction > Quadratic Edge Collapse Decimation) in order to get a manageable resolution resulting in 3D PDF files of approximately 20 MB in size for supplementary files (the final step was omitted for files uploaded to Dryad). The processed STL files were annotated and exported as 3D PDFs in Adobe Acrobat Pro DC (version 2015.006.30119) using the Tetra4D Converter plug-in (version 5.1.2). When viewing the 3D PDFs with Adobe Acrobat Reader (version 8 or higher), trusting the document by clicking on the image will activate the interactive 3D-mode and allows rotating, moving and zooming into the 3D model.

To generate the coloured 3D PDF of the mouthparts, we first segmented each mouthpart (maxillae, labium, labrum) independently and labelled each with a different colour. A surface mesh of the combined segmentation data was then generated using Generate Surface function in Amira with Unconstrained Smoothing (Smoothing Extent set to 1.5). We exported the surface data into Open Inventor Format, where it was converted to U3D format using IvTuneViewer plugged in Amira. Finally, the 3D PDF was generated by importing the U3D file to Adobe Acrobat Pro DC (version 2015.006.30119) with Tetra4D plugged in (version 5.1.2).

## Data availability

All specimens used in this study have been databased and the data is freely accessible on AntWeb (http://www.antweb.org). Each specimen can be traced by a unique specimen identifier attached to its pin (e.g. CASENT0764125). The Cybertype datasets provided in this study consist of the full micro-CT original volumetric datasets, 3D PDFs, 3D rotation video files, all light photography montage images, and all image plates including all important images of 3D models for each species. In addition to the cybertype datasets, we also provide high-resolution 3D videos and/or 3D PDFs of the mouthparts and musculature reconstructions, as well as the full micro-CT original volumetric dataset of the mouthpart scan. All data have been archived and are freely available from the Dryad Digital Repository (Hita Garcia et al. 2017c, http://dx.doi.org/10.5061/dryad.4s3v1). In addition to the cybertype data at Dryad, we also provide freely accessible 3D surface models of type material on Sketchfab (https://sketchfab.com/arilab).

## Results

### Taxonomy of *Zasphinctus* in the Afrotropical region

#### Notes on the genus in the region

At the beginning of our study we encountered a situation in which the only two valid species from the region were described from males from West Africa (Santschi 1915), whereas the material available to us consisted of three worker-based species not associated to any males. A DNA-based association is currently not possible since the two male-based species are only known from their respective type material, thus not available for any molecular analysis. There are additional males available from Nigeria and Uganda, but they are also not associated with any workers and their conspecificity with the other male-based species is uncertain. Since *Zasphinctus* is one of the rarest ant genera in the region, it is not likely that more specimens than currently available will be collected anytime soon, which means that the lack of male-worker association problems will remain. Furthermore, the two male-based species were collected in relatively arid savannah areas in West Africa while two of the worker-based species are from humid equatorial rainforests. The third worker-based species is from a savannah in Mozambique, thus geographically distant from the two male-based species.

These discrepancies led us to describe the three worker-based species independently from the already known male-based species and create a comprehensive worker-based taxonomic system for the genus in the Afrotropical region. With this approach, we follow Wilson (1964) who suggested temporarily ignoring the male-based names and establishing a sound worker-based taxonomy until males are found together with workers and the different taxonomic names can be harmonised. More recent authors concur with that approach and also opine that male-based names will eventually be matched with worker-based names using molecular data (Jaitrong and Yamane 2011; Liu et al. 2015; Staab 2015).

#### Diagnosis of Afrotropical *Zasphinctus* (workers)

The following diagnosis is based on Borowiec (2016) with modifications and additions to encompass just the Afrotropical species:

HEAD: Antennae with 12 segments and relatively short (SI 47–57), far from approaching posterior head margin. Apical antennal segment conspicuously enlarged, longer than two preceding segments combined. Head distinctly longer than broad (CI 78–86). Clypeus with cuticular apron. Lateroclypeal teeth absent. Parafrontal ridges present and well developed. Torulo-posttorular complex vertical. Antennal scrobes absent. Labrum with median notch or concavity. Proximal face of stipes not projecting beyond inner margin of sclerite, prementum exposed when mouthparts fully closed, even though only slightly so. Maxillary and labial palps 3-segmented (see section on mouthparts below). Mandibles elongate triangular, masticatory margin with 4 or 5 small denticles on basal half, denticles usually strongly reduced and inconspicuous. Eyes and ocelli absent. Head capsule with weakly to well differentiated vertical posterior surface above occipital foramen. Ventrolateral margins of head without lamella or ridge extending towards mandibles and beyond carina surrounding occipital foramen. Posterior head corners dorsolaterally immarginate. Carina surrounding occipital foramen ventrally present.

MESOSOMA: Mesosoma in profile relatively low and elongate to moderately high and stocky (LMI 34–41). In dorsal view usually slightly more than twice as long as broad (DMI2 49–58). Pronotal flange separated from collar by distinct ridge. Promesonotal connection with suture completely fused. Pronotomesopleural suture absent. Mesometapleural groove not impressed or weakly impressed. Transverse groove dividing mesopleuron absent. Pleural endophragmal pit concavity present, weakly to well developed. Mesosoma dorsolaterally immarginate. Metanotal depression or groove on mesosoma absent. Propodeal spiracle situated low on sclerite. Propodeal declivity with distinct dorsal edge or margin and rectangular in posterior view. Metapleural gland without bulla visible through cuticle. Propodeal lobes present and well developed.

LEGS: Mesotibia with single pectinate spur. Metatibia with single pectinate spur. Metabasitarsus not widening distally, circular in cross-section. Posterior flange of hind coxa not produced as raised lamella. Metatibial gland an oval patch of whitish cuticle. Metabasitarsal gland absent. Pretarsal claws of metatibia simple. Metafemur short to moderately long (MFI 75–100).

METASOMA: Abdominal segment II (petiole) sessile without peduncle and petiolar node well developed. In profile petiolar tergum between 1.0 to 1.2 times longer than high (LPI 102–123). Petiole anterodorsally marginate, dorsolaterally rounded, and laterally above spiracle weakly marginate. Laterotergites well developed and clearly demarcated. Sternum of petiole well developed with strongly anteroventrally projecting subpetiolar process, process with or without fenestra. Helcium axial and in relation to tergosternal suture placed at posttergite. Prora simple, not delimited by carina. Prora forming a U-shaped margin with median ridge. Spiracle openings of abdominal segments IV–VI circular. Abdominal segment III anterodorsally immarginate and dorsolaterally immarginate. In profile view abdominal segment III distinctly larger than succeeding segment IV, in dorsal view abdominal segment III longer than segment IV. Cinctus of abdominal segment IV not impressed. Girdling constrictions of segments IV, V, VI present and distinct, either unsculptured or cross-ribbed. Abdominal tergite IV not folding over sternite, and anterior portions of sternite and tergite equally well visible in lateral view. Pygidium large, with weakly impressed medial field.

SETATION: Most of body with numerous short to moderately long, appressed to suberect (very rarely erect) setae. Pygidium armed with modified, thick, and often peg-like setae. Hypopygium armed with modified setae.

COLOURATION: All known species predominantly dark brown to black with often lighter appendages.

#### Synoptic list of Afrotropical *Zasphinctus*

*Zasphinctus chariensis* Santschi, 1915 * [Chad]

*Zasphinctus sarowiwai* Hita Garcia sp. n. [Cameroon, Democratic Republic of Congo, Ghana, Ivory Coast, Uganda]

*Zasphinctus obamai* Hita Garcia sp. n. [Kenya]

*Zasphinctus rufiventris* Santschi, 1915 * [Benin, Mali]

*Zasphinctus wilsoni* Hita Garcia sp. n. [Mozambique]

* Only known from males and not treated in this study.

#### Diagnostic treatment

Based on a thorough examination of external morphology and character evaluation, we provide the following character matrix (Table 3) that contains 24 morphological characters of high diagnostic value.

**Table 3.** Character matrix showing all diagnostic characters used for worker-based species delimitation system of Afrotropical *Zasphinctus*.

Species	*Z. obamai*	*Z. sarowiwai*	*Z. wilsoni*
**Head in profile**	appearing longer and thinner (Fig. 4A)	appearing shorter and thicker (Fig. 4B)	appearing longer and thinner (Fig. 4C)
**Clypeal area**	without conspicuous median tooth (Fig. 4D)	with conspicuous median tooth (Fig. 4E)	without conspicuous median tooth (Fig. 4F)
**Parafrontal ridges**	dorsal outline irregularly convex and conspicuously thickened (Fig. 4G)	dorsal outline regularly convex and not conspicuously thickened (Fig. 4H)	dorsal outline mostly regularly convex and conspicuously thickened (Fig. 4I)
**Torulo-posttorular complex in dorsal view**	comparatively thicker and shorter (Fig. 4G)	comparatively thinner and longer (Fig. 4H)	comparatively thicker and shorter (Fig. 4I)
**Antennal scapes**	scape thicker: 2.2 to 2.4 times longer than broad at apex (SI2 215–242) (Fig. 4J)	scape moderately thick: 2.4 to 2.6 times longer than broad at apex (SI2 238–261) (Fig. 4K)	scape thinner: 2.7 times longer than broad at apex (SI2 267) (Fig. 4L)
**Vertex**	vertexal margin and posterior face of head strongly developed (Fig. 4M)	vertexal margin and posterior face of head weakly developed (Fig. 4N)	vertexal margin and posterior face of head strongly developed (Fig. 4O)
**Occipital margin in posterodorsal view**	outline sharp and irregularly defined (Fig. 4M)	outline sharp and very regularly defined (Fig. 4N)	outline weakly and irregularly defined (Fig. 4O)
**Occiput in posterior view**	posterior and ventral margins similarly broad; ventral margin medially protruding (Fig. 4P)	more ellipsoid; posterior and ventral margins similarly broad; ventral margin not medially protruding (Fig. 4Q)	posterior clearly broader than ventral margin; ventral margin weakly medially protruding (Fig. 4R)
**Occipital margin in posteroventral view**	outline sharp and irregularly defined (Fig. 5A)	outline sharp and very regularly defined (Fig. 5B)	outline moderately sharp and irregularly defined (Fig. 5C)
**Hypostoma**	less diverging with relatively thin and mostly rounded lateral arms (Fig. 5D)	strongly diverging with very thick and strongly rounded lateral arms (Fig. 5E)	strongly diverging with moderately thick and strongly angulate lateral arms (Fig. 5F)
**Mesosoma in profile**	relatively lower and elongate (LMI 34–36) (Fig. 5G)	moderately higher and compact (LMI 40–41) (Fig. 5H)	relatively lower and elongate (LMI 37) (Fig. 5I)
**Pleural endophragmal pit**	weakly developed and shallow but visible (Fig. 5G)	strongly developed and deep (Fig. 5H)	very weakly developed and inconspicuous (Fig. 5I)
**Mesosoma dorsal**	appearing thinner (DMI 38–40; DMI2 49–53) (Fig. 5J)	appearing thicker (DMI 41–44; DMI2 53–58) (Fig. 5K)	appearing intermediate (DMI 40; DMI2 53) (Fig. 5L)
**Petiolar tergum in profile**	relatively lower: 1.2 times longer than high (LPI 117–123) (Fig. 5M)	relatively higher: 1.0 to 1.1 times longer than high (LPI 102–112) (Fig. 5N)	relatively higher: 1.1 times longer than high (LPI 112) (Fig. 5O)
**Subpetiolar process of petiolar sternum in profile**	with thickened anterior and ventral margins and well developed concavity with differentiated fenestra (Fig. 5M)	with thickened anterior and ventral margins and well developed concavity with differentiated fenestra (Fig. 5N)	with thickened anterior and ventral margins and weak concavity without differentiated fenestra (Fig. 5O)
**Petiolar tergum in dorsal view**	relatively thinner: around 1.2 times longer than broad (DPI 82–85) (Fig. 5P)	relatively thicker: around 1.0 to 1.1. times broader than long (DPI 101–111) (Fig. 5Q)	relatively thinner: around 1.1 times longer than broad (DPI 93) (Fig. 5R)
**Subpetiolar process in ventral view**	forklike, ventral margin very thick and short (Fig. 6A)	forklike, ventral margin moderately thick and short (Fig. 6B)	forklike, ventral margin thin and long (Fig. 6C)
**Abdominal segment III in dorsal view**	appearing more trapezoidal with anterior margin more angulate (Fig. 6D)	appearing more rounded with anterior margin usually more rounded (Fig. 6E)	appearing more trapezoidal with anterior margin more angulate (Fig. 6F)
**Abdominal segment III in ventral view**	comparatively thinner, longer, and only gently narrowing towards prora (Fig. 6G)	comparatively broad, short and strongly narrowing towards prora (Fig. 6H)	comparatively broad, short and moderately narrowing towards prora (Fig. 6I)
**Posterior end of abdominal segment III in ventral view**	with thick, deep, sharply and irregularly outlined transverse groove (Fig. 6G)	with thinner, deep, sharply and relatively regularly outlined transverse groove (Fig. 6H)	transverse groove absent, instead with irregular grooves and rugosity (Fig. 6I)
**Prora in anteroventral view**	well-developed with thick, irregularly shaped and rounded lateroventral margins (Fig. 6J)	well-developed with sharply and very regularly shaped lateroventral margins (Fig. 6K)	very weak to almost absent lateroventral margins (Fig. 6L)
**Abdominal segment VI in dorsal view**	distinctly longer: 1.7 times broader than long (DA6I 163–173) (Fig. 6M)	distinctly shorter: around 1.9 to 2 times broader than long (DA6I 186–197) (Fig. 6N)	distinctly longer: 1.6 times broader than long (DA6I 159) (Fig. 6O)
**Girdling constrictions abdominal segments IV, V, VI**	unsculptured (Fig. 6P)	cross-ribbed, much weaker on IV than V & VI (Fig. 6Q)	unsculptured (Fig. 6R)
**General surface sculpture**	mostly smooth and shining with abundant, relatively deep piliferous punctures, except for reticulate-punctate anteromedian area of cephalic dorsum, anterior pronotum, mesopleuron, lateral propodeum, most of lateral petiole, and hypopygium	almost completely smooth and very shining with scattered, relatively deep piliferous punctures; sometimes with punctate sculpture on metapleuron	cephalic dorsum mostly reticulate-rugose, mesosoma and petiole laterally mostly reticulate-punctate, hypopygium reticulate-rugose, remainder of body predominanly smooth and shining with abundant piliferous punctures

#### Identification key to Afrotropical *Zasphinctus* species (workers)

**Table:** 

1	With head in full-face view median clypeal area with conspicuous tooth (Fig. 4E, H) and torulo-posttorular complex comparatively long (Fig. 4H); in posterodorsal view vertexal margin and posterior face of head weakly developed (Fig. 4N)	***Z. sarowiwai***
–	With head in full-face view median clypeal area without any tooth (Fig. 4D, 4F, G, I) and torulo-posttorular complex comparatively short (Fig. 4G, I); in posterodorsal view vertexal margin and posterior face of head strongly developed (Fig. 4M, O)	2
2	With head in full-face view parafrontal ridges with irregularly shaped dorsal outline (Fig. 4G); petiolar tergum in profile relatively lower, ca. 1.2 times longer than high (LPI 117–123) (Fig. 5M)	***Z. obamai***
–	With head in full-face view parafrontal ridges with regularly shaped dorsal outline (Fig. 4I); petiolar tergum in profile relatively higher, ca. 1.1 times longer than high (LPI 112) (Fig. 5O)	***Z. wilsoni***

#### Zasphinctus obamai

Taxon classificationAnimaliaHymenopteraFormicidae

Hita Garcia sp. n.

http://zoobank.org/2B973F61-641C-436D-89AC-5955B519563A

Figs 3 , 4A, D, G, J, M, P , 5A, D, G, J, M, P , 6A, D, G, J, M, P , 7 , 8 , 13A , 16, Video 1

##### Type material.

**Holotype**, pinned worker, KENYA, Western Province, Kakamega Forest, Buyangu, 0.35222, 34.8647, 1640 m, secondary rainforest, leaf litter, collection code FHG00001, VII.-VIII.2004 (*F. Hita Garcia*) (NMKE: CASENT0764125). **Paratypes**, seven pinned workers: two with same data as holotype (BMNH: CASENT0764126; MCZC: CASENT0764127); one from KENYA, Western Province, Kakamega Forest, Isecheno, equatorial rainforest, sifted litter and soil under *Morus mesozygia*, 0.34, 34.85, 1550 m, 6.XI.2002 (*W. Okeka*) (LACM: CASENT0178218; ZFMK: CASENT0764648); two from KENYA, Western Province, Kakamega Forest, Kisere Forest Fragment, 0.38505, 34.89378, 1650 m, rainforest, ex leaf litter, Transect 11, collection code FHG00036, 16.VII.2007 (*F. Hita Garcia*) (NMKE: CASENT0764128; NMKE: CASENT0764129); and one from KENYA, Western Province, Kakamega Forest, Bunyala Forest Fragment, 0.37889, 34.69917, 1448 m, Winkler leaf litter extraction, collection code ANTC39476, VIII.2008 (*G. Fischer*) (ZFMK: CASENT0764647).

**Figure 3.**
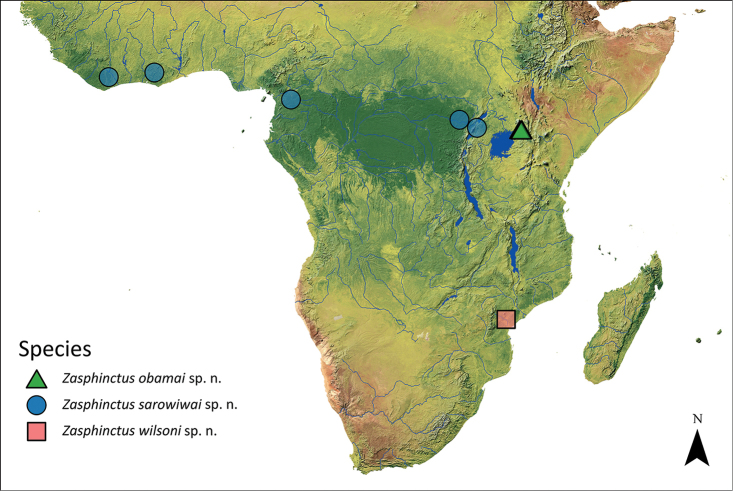
Map of sub-Saharan Africa showing the known distribution of the *Z. obamai* sp. n., *Z. sarowiwai* sp. n., and *Z. wilsoni* sp. n.

**Cybertypes**, the cybertype dataset consists of all volumetric raw data in DICOM format, 3D PDFs and 3D rotation videos of scans of head, mesosoma, metasoma, and the full body of the physical holotype (NMKE: CASENT0764125) and/or one paratype (MCZC: CASENT0764127) in addition to montage photos illustrating head in full-face view, profile and dorsal views of the body of both specimens. The data is deposited at Dryad and can be freely accessed as virtual representation of both types (Hita Garcia et al. 2017c, http://dx.doi.org/10.5061/dryad.4s3v1). In addition to the cybertype data at Dryad, we also provide a freely accessible 3D surface model of the holotype at Sketchfab (https://sketchfab.com/models/dfe15a58514c4be89cdeff7f9713091c).

**Video 1.** 3D rotation video of *Zasphinctus obamai* sp. n. holotype worker (CASENT0764125) based on shaded volumetric surface rendering of full body.

##### Differential worker diagnosis.

See Table 3.

##### Worker measurements and indices.

See Table 4.

**Table 4.** Morphometric data of the three species treated in this study.

	*Z. obamai* (N=6)			*Z. sarowiwai* (N=11)			*Z. wilsoni* (N=1)
	Min	Max	Mean	Min	Max	Mean	
**HL**	0.55	0.59	0.56	0.78	0.90	0.86	0.60
**HW**	0.44	0.47	0.45	0.64	0.77	0.73	0.49
**SL**	0.26	0.31	0.28	0.41	0.50	0.48	0.32
**SW**	0.12	0.14	0.13	0.17	0.21	0.19	0.12
**PH**	0.26	0.29	0.27	0.44	0.52	0.49	0.32
**PW**	0.28	0.33	0.30	0.47	0.55	0.52	0.35
**DML**	0.53	0.65	0.59	0.85	0.99	0.95	0.66
**WL**	0.73	0.81	0.77	1.08	1.30	1.22	0.87
**MFL**	0.33	0.37	0.35	0.58	0.69	0.64	0.49
**PTL**	0.27	0.29	0.28	0.40	0.47	0.44	0.29
**PTH**	0.22	0.24	0.23	0.39	0.45	0.42	0.26
**PTW**	0.23	0.26	0.24	0.41	0.50	0.47	0.27
**A3L**	0.33	0.39	0.36	0.50	0.59	0.55	0.48
**A3W**	0.38	0.43	0.41	0.56	0.67	0.63	0.43
**A4L**	0.26	0.29	0.28	0.41	0.56	0.50	0.31
**A4W**	0.46	0.52	0.49	0.71	0.83	0.79	0.54
**A5L**	0.25	0.29	0.27	0.40	0.49	0.45	0.32
**A5W**	0.47	0.52	0.49	0.71	0.85	0.80	0.55
**A6L**	0.26	0.30	0.28	0.36	0.41	0.39	0.32
**A6W**	0.45	0.49	0.47	0.67	0.78	0.73	0.51
**CI**	78	80	80	82	86	84	82
**SI**	47	53	50	53	57	55	53
**SI2**	215	242	228	238	261	247	267
**DMI**	38	40	39	41	44	42	40
**DMI2**	49	53	51	53	58	55	53
**LMI**	34	36	36	40	41	40	37
**MFI**	75	79	77	88	91	89	100
**LPI**	117	123	120	102	112	105	112
**DPI**	82	93	88	101	111	105	93
**DA3I**	108	115	112	112	117	114	90
**DA4I**	170	181	176	145	173	159	174
**DA5I**	174	188	180	167	181	177	172
**DA6I**	163	173	169	186	197	189	159

##### Etymology.

This species is named in honour of Barack Hussein Obama, the 44th President of the United States of America. We want to acknowledge his important efforts undertaken for the conservation of fragile natural habitats around the globe. Also, the type locality of *Z. obamai* is geographically close to the hometown of Obama’s paternal family in Western Kenya.

##### Distribution and biology.

*Zasphinctus obamai* is only known from the type locality, the Kakamega Forest in Western Kenya, which is a tropical equatorial rainforest. Despite a thorough ant inventory (Hita Garcia et al. 2009), *Z. obamai* was only sampled four times making it one of the rarest ant species of the Kakamega Forest. It was only found in the leaf litter layer of primary or near-primary forest habitats. Considering the rarity of this species in the type locality it is possible that it might also be encountered in other rainforest localities westwards of Kakamega, but eluded collections in the past. However, presently, *Z. obamai* appears to be endemic to this one forest.

##### Diagnostic comments.

*Zasphinctus obamai* appears to be morphologically closer to *Z. wilsoni* than to *Z. sarowiwai*. Among other important differences, *Z. obamai* and *Z. wilsoni* are significantly smaller, lack a median clypeal tooth, and have a clearly defined vertexal margin compared to *Z. sarowiwai*. *Zasphinctus obamai* and *Z. wilsoni* can be easily separated by the characters provided above in Table 3. On the basis of the type series, there is no observable intraspecific variation.

**Figure 4.**
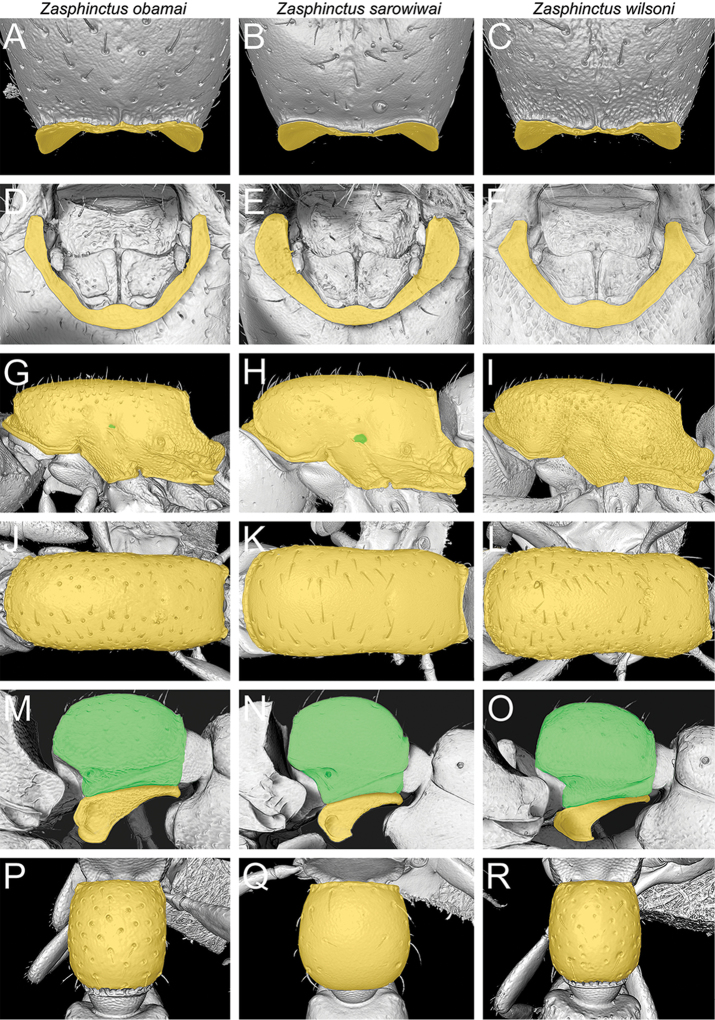
Illustrated diagnostic character matrix based on micro-CT images used for species delimitations (*Z. obamai* = left column, *Z. sarowiwai* = middle column, *Z. wilsoni* = right column). **A, B, C** Cephalic capsule in profile (virtually dissected) **D, E, F** Clypeus and torulo-posttorular complex in anterior view **G, H, I** Anterior head (antennae virtually removed) showing parafrontal ridges (orange) and torulo-posttorular complex (green) **J, K, L** Antennal scape in dorsal view (virtually dissected) **M, N, O** Head in posterodorsal view showing vertexal margin (orange), posterior face, and occipital margin (green) **P, Q, R** Head in posterior view showing occiput and occipital foramen (virtually dissected) (ventral head facing upwards).

**Figure 5.**
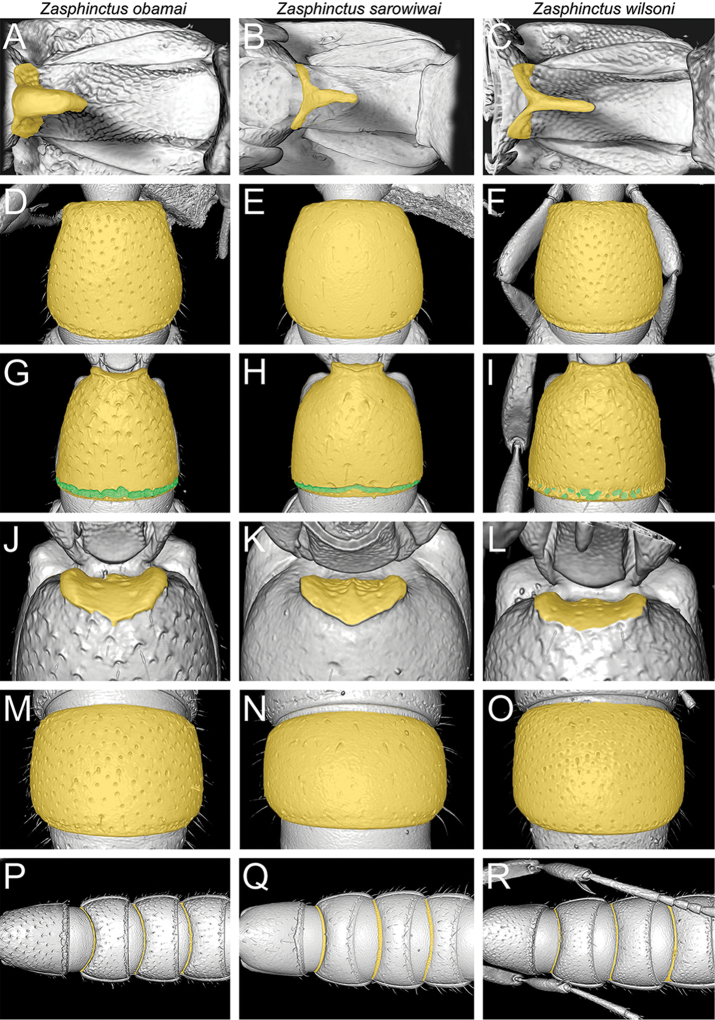
Illustrated diagnostic character matrix based on micro-CT images used for species delimitations (*Z. obamai* = left column, *Z. sarowiwai* = middle column, *Z. wilsoni* = right column). **A, B, C** Posterior head in ventral view showing ventral occipital margin (virtually dissected) **D, E, F** Head in ventral view showing mouthparts and hypostoma (virtually dissected). **G, H, I** Mesosoma in profile (orange) with pleural endophragmal pit (green) **J, K, L** Mesosoma in dorsal view **M, N, O** Petiole in profile showing petiolar tergum (green) and petiolar sternum (orange) with subpetiolar process **P, Q, R** Petiole in dorsal view.

**Figure 6.**
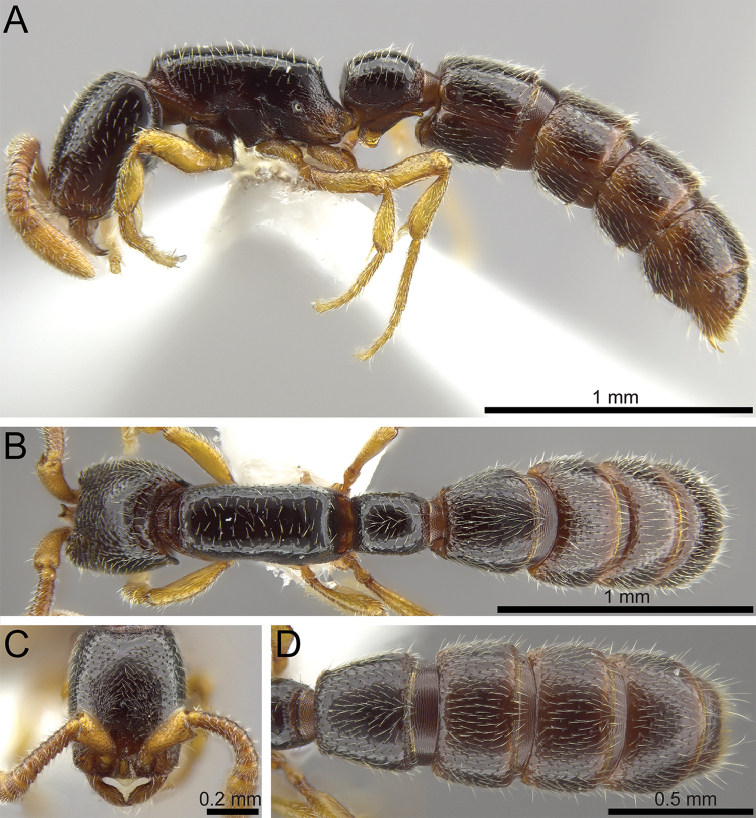
Illustrated diagnostic character matrix based on micro-CT images used for species delimitations (*Z. obamai* = left column, *Z. sarowiwai* = middle column, *Z. wilsoni* = right column). **A, B, C** Subpetiolar process of petiolar sternum in ventral view (virtually dissected) **D, E, F** Abdominal segment III in dorsal view **G, H, I** Abdominal segment III (orange) in ventral view with posterior end (green) **J, K, L** Abdominal segment III in anteroventral view showing prora (virtually dissected) **M, N, O** Abdominal segment VI in dorsal view **P, Q, R** Abdominal segments III, IV, V, and VI in ventral view showing girdling constrictions.

**Figure 7.**
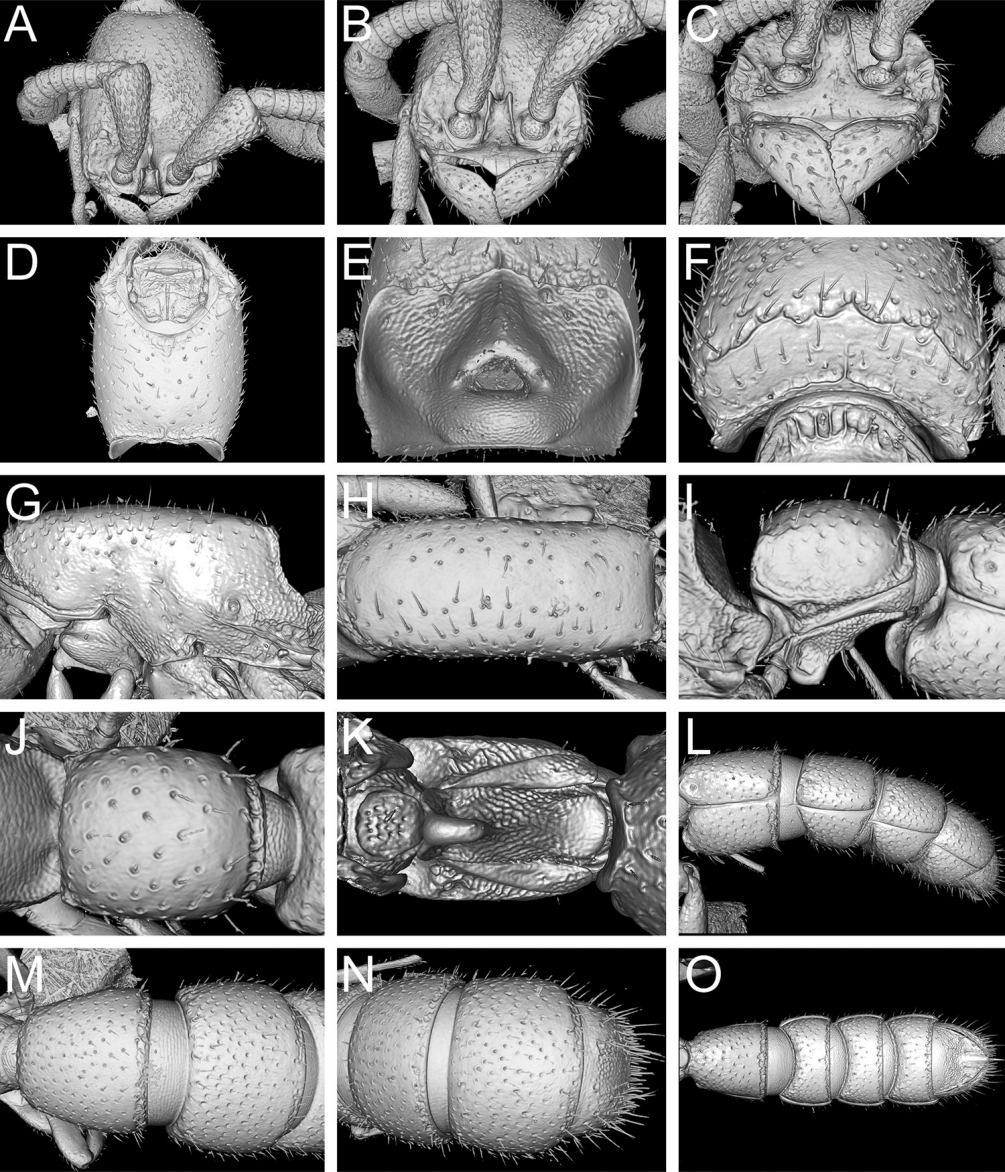
*Zasphinctus obamai* sp. n. holotype worker (CASENT0764125). **A** Body in profile **B** Body in dorsal view **C** Head in full-face view **D** Abdominal segments III–VII in dorsal view.

**Figure 8.**
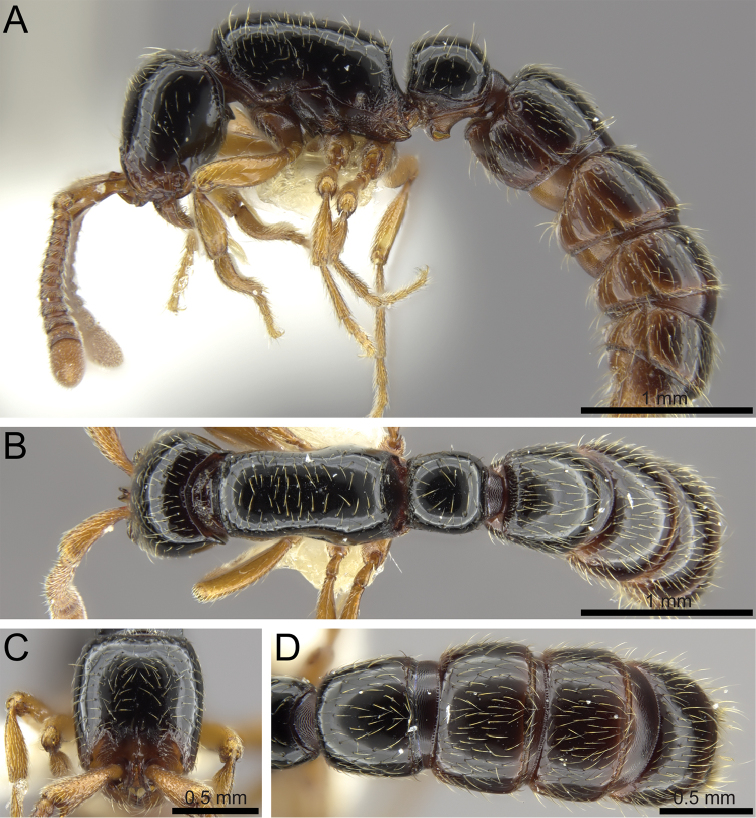
Shaded surface display volume renderings of 3D models of *Zasphinctus obamai* sp. n. paratype worker (CASENT0764127). **A** Head in full-face dorsal view **B** Head in anterodorsal view **C** Anterior cephalic dorsum and mandibles in anterodorsal view **D** Head in ventral view **E** Occiput in posterior view (ventral head facing upwards) **F** Head in posterodorsal view **G** Mesosoma in profile **H** Mesosoma in dorsal view **I** Abdominal segment II (petiole) in profile **J** Abdominal segment II (petiole) in dorsal view **K** Abdominal segment II (petiole) in ventral view **L** Abdominal segments III–VII in profile **M** Abdominal segments III and IV in dorsal view **N** Abdominal segments V–VII in dorsal view **O** Abdominal segments III–VII in ventral view.

#### Zasphinctus sarowiwai

Taxon classificationAnimaliaHymenopteraFormicidae

Hita Garcia sp. n.

http://zoobank.org/DB20AFDC-3644-44A5-AA74-9B53249B5C0D

Figs 3 , 4B, E, H, K, N, Q , 5B, E, H, K, N, Q , 6B, E, H, K, N, Q , 9 , 10 , 13B , 14 , 15, Videos 2, 4, 5

##### Type material.

**Holotype**, pinned worker, CAMEROON, Centre Province, Mbalmayo, 3.4597, 11.4714, ca. 600 m, rainforest, XI.1993 (*N. Stork*) (BMNH: CASENT0764654). **Paratypes**, three pinned workers with same data as holotype (BMNH: CASENT0764646; CASENT0764649; CASENT0764650).

**Cybertypes**, the cybertype dataset consists of all volumetric raw data in DICOM format, 3D PDFs and 3D rotation videos of scans of head, mesosoma, metasoma, and the full body of the physical holotype (BMNH: CASENT0764654) and/or one paratype (BMNH: CASENT0764650) in addition to montage photos illustrating head in full-face view, profile and dorsal views of the body of both specimens. The data is deposited at Dryad and can be freely accessed as virtual representation of both types (Hita Garcia et al. 2017c, http://dx.doi.org/10.5061/dryad.4s3v1). In addition to the cybertype data at Dryad, we also provide a freely accessible 3D surface model of the holotype at Sketchfab (https://sketchfab.com/models/3e5a54cb8ea94028a49f0722bd5eefe8).

##### Non-type material.

DEMOCRATIC REPUBLIC OF CONGO: Epulu, 1.38333, 28.58333, 750 m, rainforest, 1.XI.1995 (*S.D. Torti*); GHANA: Wiawso, 6.2158, -2.485, ca. 160 m, 25.IV.1969 (*D. Leston*); IVORY COAST: Tai Forest, 5.75, -7.12, ca. 250 m, rainforest, 18.–20.V.1977 (*T. Diomande*); UGANDA: Western, Kabarole, Kibale National Park, Kanyawara Biological Station, 0.56437, 30.36059, 1510–1520 m, rainforest, 6.–16.VIII.2012 (different independent collectors: *F.A. Esteves*, *F. Hita Garcia* & *P.G. Hawkes*).

**Figure 9.**
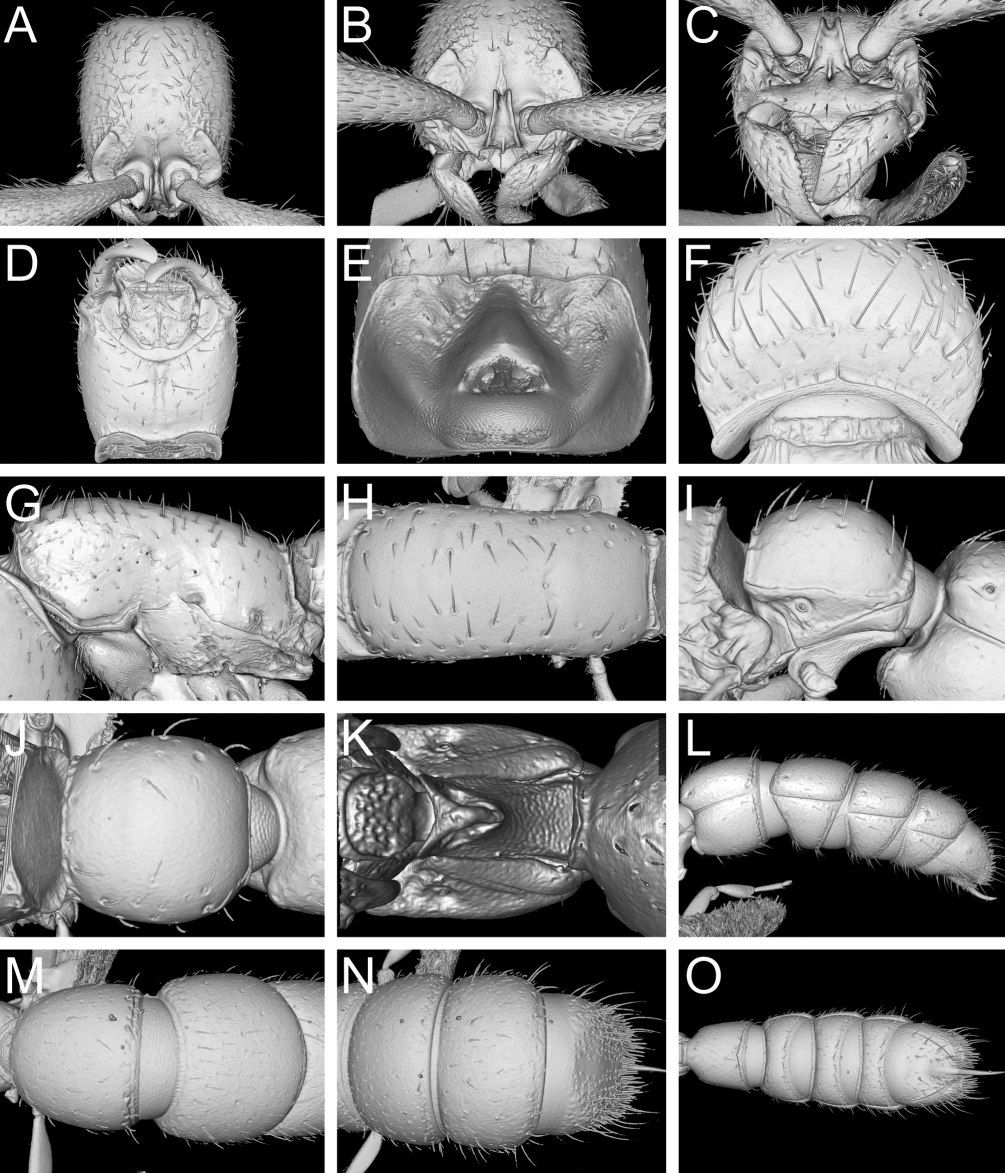
*Zasphinctus sarowiwai* sp. n. paratype worker (CASENT0764650). **A** Body in profile **B** Body in dorsal view **C** Head in full-face view **D** Abdominal segments III–VI in dorsal view.

##### Differential worker diagnosis.

See Table 3.

##### Worker measurements and indices (N=11).

See Table 4.

##### Etymology.

The name of the new species is a patronym in honour of the famous Nigerian writer, environmentalist, and human rights activist Kenule Beeson “Ken” Saro-Wiwa. By naming a species from threatened rainforest habitats after him, we want to acknowledge his environmental legacy and draw attention to the often-problematic conservation situation in most Afrotropical rainforests.

##### Distribution and biology.

The new species has a comparatively wide distribution ranging from Ivory Coast to Uganda, even though it is not known from all countries in-between. However, this is likely based on a sampling artefact considering the rarity of *Zasphinctus* in general and the poor sampling in most African countries. Therefore, we expect future collections in all countries in-between. All samples are from rainforest habitats at elevations from 250 to 1510 m. Based on the available collection data, the species lives in soil and leaf litter.

##### Diagnostic comments.

*Zasphinctus sarowiwai* differs in most diagnostic characters from the other two Afrotropical species. Most obviously, it can be separated from the other species by its much larger body size, the prominent median clypeal tooth, and the almost complete lack of surface sculpture. Despite its wide distribution range, there is very little observable variation. Most notably, the colour appears to be generally darker in the specimens from Uganda and Cameroon, which are uniformly very dark brown to black, while the specimens from West Africa tend to have a much lighter abdomen and often relatively bright legs. Furthermore, we observed some variation in the material from Uganda. In some specimens, the subpetiolar process of the petiolar sternum had a slightly weaker, but still distinct, fenestra compared to the material from other localities, and the ventral margin of the process had a posteroventral tooth-like projection. In addition, the anterodorsal margin of abdominal segment III was slightly more angulate in a few specimens while in several other specimens the metapleuron had some weak punctate sculpture. Overall, we consider this variation as geographic and very well within the intraspecific range of such a widespread species.

**Figure 10.**
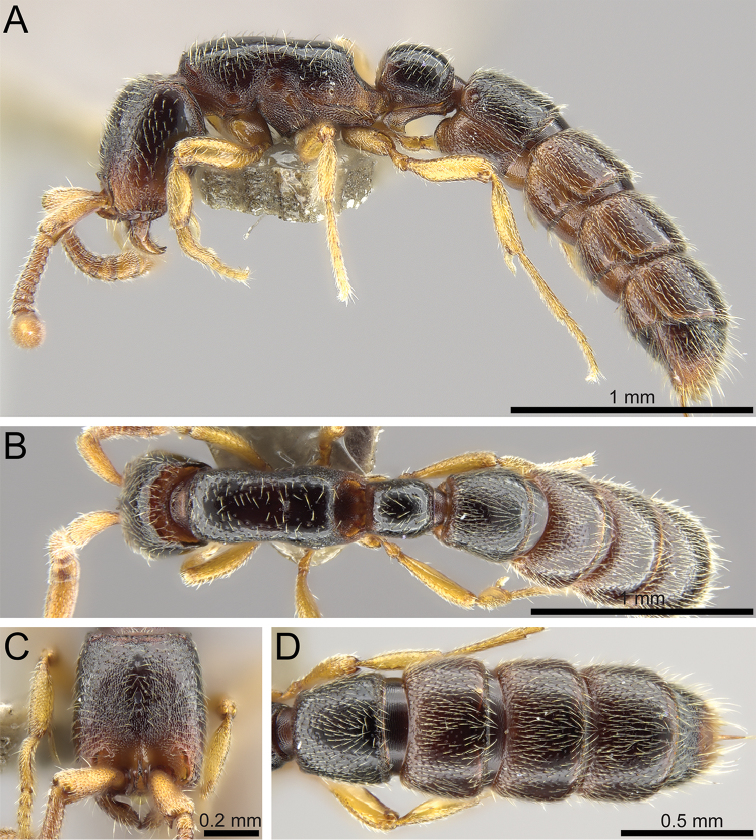
Shaded surface display volume renderings of 3D models of *Zasphinctus sarowiwai* sp. n. holotype worker (CASENT0764654). **A** Head in full-face dorsal view **B** Head in anterodorsal view **C** Anterior cephalic dorsum and mandibles in anterodorsal view **D** Head in ventral view **E** Occiput in posterior view (ventral head facing upwards) **F** Head in posterodorsal view **G** Mesosoma in profile **H** Mesosoma in dorsal view **I** Abdominal segment II (petiole) in profile **J** Abdominal segment II (petiole) in dorsal view **K** Abdominal segment II (petiole) in ventral view **L** Abdominal segments III–VII in profile **M** Abdominal segments III and IV in dorsal view **N** Abdominal segments V–VII in dorsal view **O** Abdominal segments III–VII in ventral view.

**Video 2.** 3D rotation video of *Zasphinctus sarowiwai* sp. n. paratype worker (CASENT0764650) based on shaded volumetric surface rendering of full body.

#### Zasphinctus wilsoni

Taxon classificationAnimaliaHymenopteraFormicidae

Hita Garcia sp. n.

http://zoobank.org/355B3D80-3029-4C8A-B48C-939C11914552

**Figure 11.**
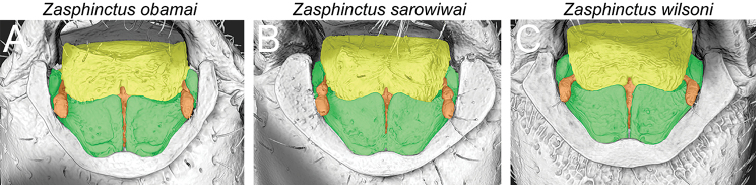
*Zasphinctus wilsoni* sp. n. holotype worker (MCZ-ENT-00512764). **A** Body in profile **B** Body in dorsal view **C** Head in full-face view **D** Abdominal segments III–VII in dorsal view.

**Figure 12.**
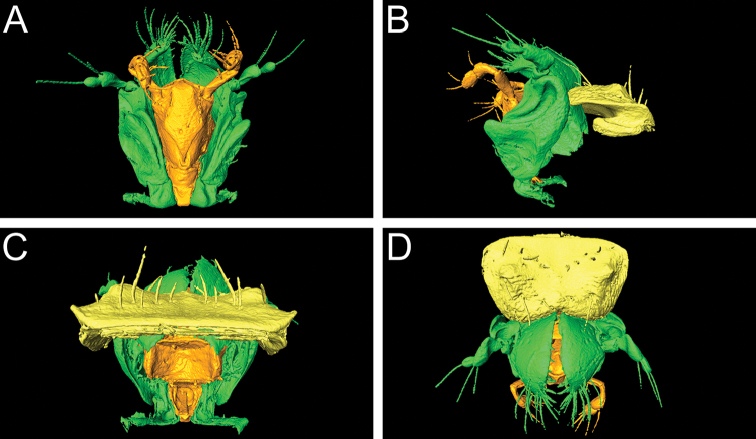
Shaded surface display volume renderings of 3D models of *Zasphinctus wilsoni* sp. n. holotype worker (MCZ-ENT-00512764). **A** Head in full-face dorsal view **B** Head in anterodorsal view **C** Anterior cephalic dorsum and mandibles in anterodorsal view **D** Head in ventral view. **E** Occiput in posterior view (ventral head facing upwards) **F** Head in posterodorsal view **G** Mesosoma in profile **H** Mesosoma in dorsal view **I** Abdominal segment II (petiole) in profile **J** Abdominal segment II (petiole) in dorsal view **K** Abdominal segment II (petiole) in ventral view **L** Abdominal segments III–VII in profile **M** Abdominal segments III and IV in dorsal view **N** Abdominal segments V–VII in dorsal view **O** Abdominal segments III–VII in ventral view.

**Figure 13.**
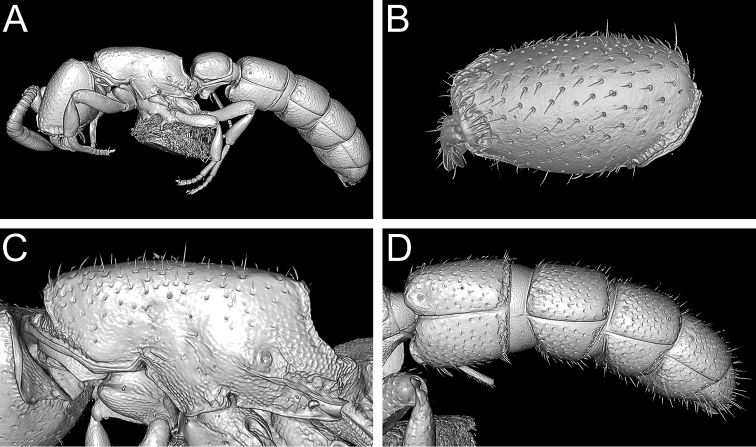
Shaded surface display volume renderings of 3D models of mouthparts (excluding mandibles) in closed configuration (green=maxillae; yellow=labrum; orange=labium). **A** *Zasphinctus obamai* sp. n. (CASENT0764127) **B** *Zasphinctus sarowiwai* sp. n. (CASENT0764654) **C** *Zasphinctus wilsoni* sp. n. (MCZ-ENT-00512764).

Figs 3 , 4C, F, I, L, O, R , 5C, F, I, L, O, R , 6C, F, I, L, O, R , 11 , 12 , 13C, Video 3

##### Type material.

**Holotype**, pinned worker, Mozambique, Sofala, Gorongosa National Park, 2 km S Chitengo, -18.99472, 34.35769, 1 m, secondary forest, leaf litter, collection code ANTC37418, 30.V.2012 (*G.D. Alpert*) (MCZC: MCZ-ENT-00512764).

**Cybertype**, the cybertype dataset consists of the volumetric raw data in DICOM format, as well as 3D PDFs and 3D rotation videos of scans of the head, mesosoma, metasoma, and the full body of the physical holotype (MCZC: MCZ-ENT-00512764) in addition to montage photos illustrating head in full-face view, profile and dorsal views of the body. The data is deposited at Dryad and can be freely accessed as virtual representation of the holotype (Hita Garcia et al. 2017c, http://dx.doi.org/10.5061/dryad.4s3v1). In addition to the cybertype data at Dryad, we also provide a freely accessible 3D surface model of the holotype at Sketchfab (https://sketchfab.com/models/36bab7ecaa8d45b18013ea679b7ca54a).

##### Differential worker diagnosis.

See Table 3.

##### Worker measurements and indices (N=1).

See Table 4.

##### Etymology.

This new species is dedicated to the renowned scientist, author, and conservationist Edward O. Wilson from Harvard University in honour of his more than six decades of accomplishments to the fields of myrmecology, sociobiology, biodiversity, and conservation.

##### Distribution and biology.

Currently, *Z. wilsoni* is only known from its type locality, the Gorongosa National Park where it was collected in the leaf litter of a secondary dry forest. Considering how generally undersampled south-eastern Africa is, it is likely that *Z. wilsoni* might be encountered in other woodland localities in Mozambique, Tanzania, or Zimbabwe.

##### Diagnostic comments.

*Zasphinctus wilsoni* is morphologically closer to *Z. obamai* than to *Z. sarowiwai*. It shares the smaller body size, the lack of median clypeal tooth, and a clearly defined vertexal margin with *Z. obamai*, separating both from *Z. sarowiwai*. However, the conspicuous surface sculpture on the cephalic dorsum and the sides of mesosoma and petiole clearly distinguishes *Z. wilsoni* from the other two species. Since *Z. wilsoni* is only known from the holotype there is no available information about intraspecific variation.

**Video 3.** 3D rotation video of *Zasphinctus wilsoni* sp. n. holotype worker (MCZ-ENT-00512764) based on shaded volumetric surface rendering of full body.

### 3D mouthparts morphology (excluding mandibles)

**Figure 14.**
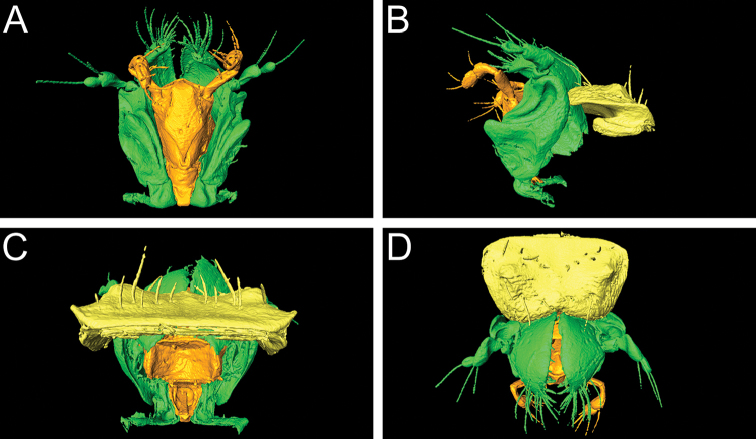
Volumetric 3D model of segmented surface reconstructions of the mouthparts of *Zasphinctus sarowiwai* sp. n. (CASENT0764652) in open configuration (green=maxillae; yellow=labrum; orange=labium). **A** Frontal view **B** Lateral view **C** Posterior view **D** Dorsal view.

The small number and the preservation conditions of the specimens available for this study provided some limitations for the examination of mouthparts. It was not possible to dissect in vivo or micro-CT scan the open mouthparts of *Z. obamai*, nor of *Z. wilsoni*. Fortunately, the mouthparts of one pinned specimen of *Z. sarowiwai* were open and mostly exposed, thus available for superficial examination under the light microscope and for micro-CT scanning. Consequently, we were unable to test mouthpart morphology in detail for species delimitation. However, based on the limited information observable in closed condition, there appears to be no significant difference between the three species (Fig. 13). In the following we briefly describe the open mouthparts of *Z. sarowiwai* based on a 3D reconstruction of segmented micro-CT data (Fig. 14 and Video 4):

*Labrum*: distal margin conspicuously cleft medially; median area from anterior cleft to proximal articulation very thin, dividing labrum into two lobes; each lobe bulging medially; lateroventrally with two conspicuous hook-like labral arms projecting parallel to remainder of labrum; row of ten to twelve setae (1 very long pair plus four/five shorter pairs) on basal third of exterior face; row of four to six setae (1 very long pair plus one/two shorter pairs) on exterior face close to distal margin; labral tubercles absent.

*Maxillae*: maxillary palp three-segmented with second segment being greatly enlarged, third segment with very long seta, second with two long setae; deep and conspicuous diagonal, transverse stipital groove present dividing stipes into proximal external face and distal external face; articulation of labrum with maxillae of labro-stipital type via lateral extension/shoulder; proximal faces projecting beyond inner margin of stipites, thus almost completely concealing prementum; galea with well-developed galeal crown and maxillary brush, galeal comb apparently absent; lacinial comb not observable.

*Labium*: labial palp three-segmented with first segment being greatly enlarged, first and second segment with one long seta, third segment with three long setae; premental shield with several moderately long setae; shape of glossa not observable (structure collapsed); subglossal brush present and conspicuous with numerous long and thick setae; paraglossae absent.

**Video 4.** 3D rotation video of segmented surface reconstructions of the mouthparts of *Zasphinctus sarowiwai* sp. n. (CASENT0764652) in open configuration (green= maxillae; yellow=labrum; orange=labium).

## Cuticle thickness

The results of our cuticle thickness data are provided in Table 5. The measurements show absolute values of 13–22 µm in *Z. obamai*, 20–30 µm in *Z. sarowiwai*, and 14–21 µm in *Z. wilsoni*, and by putting these in relation to body size the thickness indices range between 31–44. On the basis of cuticle thickness data throughout nine subfamilies of ants (Peeters et al. 2017), the cuticle thickness values of our three *Zasphinctus* species are at the upper range meaning that these species possess among the thickest cuticles measured so far.

**Table 5.** Morphometric data generated from 3D measuring cuticle thickness. For each species the five raw measurements with corresponding calculations into indices are given, as well as mean values and standard deviations (SD).

Species	*Z. obamai*		*Z. sarowiwai*		*Z. wilsoni*	
	in mm	**CCCI**	in mm	**CCCI**	in mm	**CCCI**
CCC 1	0.019	41	0.022	30	0.014	29
CCC 2	0.018	41	0.023	32	0.014	29
CCC 3	0.019	41	0.022	29	0.016	32
CCC 4	0.021	47	0.024	33	0.017	34
CCC 5	0.022	49	0.023	31	0.015	30
MEAN	0.019	**44**	0.023	**31**	0.015	**31**
SD	0.001	3	0.001	1	0.001	2
	in mm	**PRCI**	in mm	**PRCI**	in mm	**PRCI**
PRC 1	0.017	37	0.027	36	0.018	38
PRC 2	0.017	38	0.027	36	0.020	40
PRC 3	0.016	36	0.025	34	0.019	39
PRC 4	0.016	35	0.029	39	0.020	41
PRC 5	0.015	34	0.030	40	0.021	42
MEAN	0.016	**36**	0.027	**37**	0.020	**40**
SD	0.001	1	0.002	2	0.001	1
	in mm	**ASIICI**	in mm	**ASIICI**	in mm	**ASIICI**
ASIIC 1	0.013	29	0.025	33	0.017	34
ASIIC 2	0.014	31	0.026	35	0.019	39
ASIIC 3	0.016	35	0.027	37	0.020	41
ASIIC 4	0.013	29	0.026	35	0.020	42
ASIIC 5	0.014	31	0.030	40	0.020	40
MEAN	0.014	**31**	0.027	**36**	0.019	**39.2**
SD	0.001	2	0.002	2	0.001	3
	in mm	**ASIIICI**	in mm	**ASIIICI**	in mm	**ASIIICI**
ASIIIC 1	0.013	29	0.022	30	0.018	37
ASIIIC 2	0.015	33	0.029	39	0.018	37
ASIIIC 3	0.013	29	0.021	29	0.017	34
ASIIIC 4	0.016	36	0.022	30	0.017	35
ASIIIC 5	0.013	30	0.020	28	0.019	38
MEAN	0.014	**31**	0.021	**31**	0.018	**36**
SD	0.001	3	0.003	4	0.001	1

### Thoracic and abdominal musculature

**Figure 15.**
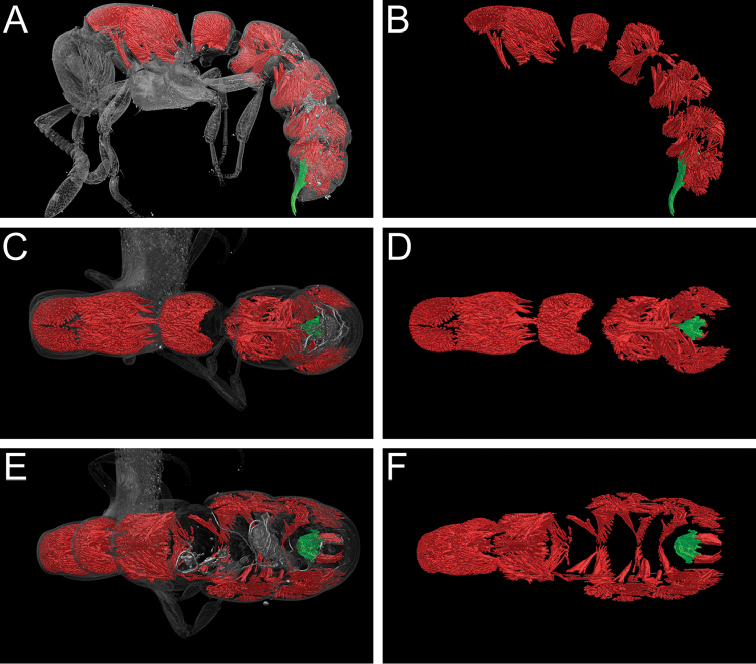
Still images of 3D model of full body of *Zasphinctus sarowiwai* sp. n. holotype worker (CASENT0764654). False-colour volume rendering of segmented mesosoma and metasoma musculature (red) and sting apparatus (green) superimposed on semi-transparent surface model (**A, C, E**) or stand-alone (**B, D, F**). **A, B** Body in profile view **C, D** Body in dorsal view **E, F** Body in posterodorsal view.

Based on our virtually reconstructed and segmented data, we can show that the mesosoma and metasoma both contain high degrees of musculature (Fig. 15; Video 5). The propodeum is tightly packed with dorsal and ventral muscles moving the abdominal segment II (petiole) and stabilizing the weight of the abdominal segments III to VII. Due to the massive size of the latter, the volume of the propodeal muscles attaching to the anterior petiole is high and comparable to that of the neck muscles in the pronotum. The petiole and the following segments also have a high muscle density, which is prominently visible in lateral (Fig. 15A, 15B), dorsal (Fig. 15C, 15D), and posterodorsal views (Fig. 15E, 15F) in the segmented 3D models of the metasoma. While the muscles in abdominal segment II – and to a lesser extent in segment III – evenly fill almost the entire segment, those in segments IV to VII are mostly limited to positions along the lateral and ventral walls. Finally, attached to the sting apparatus are two separate muscle sets, the protractors and retractors of the sting shaft. The former set is responsible for extending the sting from the tip of the abdomen during attack or defence and the latter for retracting it back to its resting position within the abdomen (visible in dorsal view in Fig. 15F).

## Discussion

### Virtual recovery of morphology

**Figure 16.**
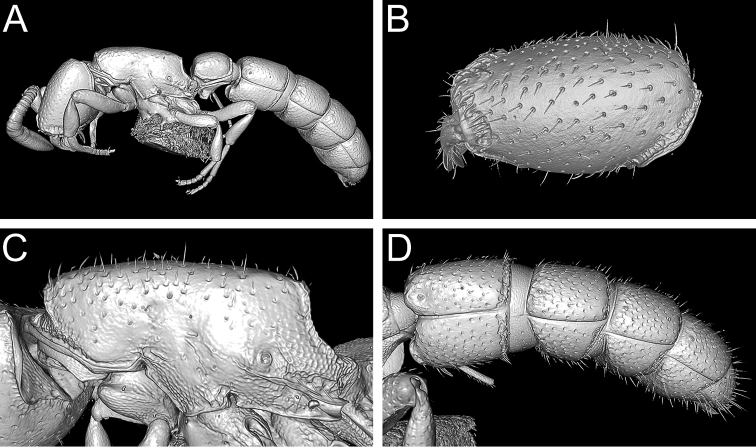
Comparison of full body scan versus single body part scans based on *Zasphinctus obamai* sp. n. holotype (CASENT0764125) and paratype worker (CASENT0764127). **A** Full body scan (CASENT0764125) **B** Scan of head (CASENT0764125) **C** Scan of mesosoma (CASENT0764127) **D** Scan of metasoma showing abdominal segments III to VII in profile (CASENT0764127).

Almost all previous studies that used micro-CT for invertebrate taxonomy encountered problems with the achievable voxel resolution in relation to body size resulting in a poor recovery of certain, very fine or small structures, such as setae, ommatidia, and microsculpture (Faulwetter et al. 2013, 2014; Fernández et al. 2014; Carbayo et al. 2016; Fischer et al. 2016). In the case of ant taxonomy this was intensively discussed by Hita Garcia et al. (2017a) who achieved voxel sizes of around 5 µm for full body scans of the two treated species. Based on these results and in order to improve the voxel resolution and present better resolved 3D reconstructions, we scanned the head, the mesosoma, and the metasoma separately in addition to a full body scan for each species. As a consequence, we attained smaller voxel sizes for the 3D models of the single body parts (0.95–2.83 µm versus 3.00–4.61 µm) resulting in a much higher resolution, and significantly reduced or eliminated the problems encountered by Hita Garcia et al. (2017a) (Fig. 16), except for ommatidia that are absent in *Zasphinctus* workers. While setae were poorly recovered by Hita Garcia et al. (2017a), they are very well visible in our 3D models of single body parts presented in this study. However, due to the higher voxel size, our full body scans of the *Zasphinctus* species have a weaker resolution of setae. Furthermore, compared to the physical specimens, surface sculpture was recovered with high morphological accuracy in the 3D models of single body parts, whereas it was only poorly noticeable in the full body scans. Surprisingly, fine surface sculpture on some body parts was even more observable in the 3D models than in the physical material, due to a limited magnification of our light microscope to 100 ×.

**Video 5.** 3D rotation video of full body of *Zasphinctus sarowiwai* sp. n. holotype worker (CASENT0764654). False-colour volume rendering of segmented mesosoma and metasoma musculature (red) and sting apparatus (green) superimposed on semitransparent surface model.

The visualised reconstruction of the mouthparts provides a comparatively adequate and detailed 3D model of the maxillae, labium, and labrum, but also presents some important limitations. The general morphology of maxillae, labium and labrum are well recovered, and they are very similar to the mouthparts of *Z. steinheili* that were described by Gotwald (1969). The vast majority of setae are well visible, as is the surface sculpture of most structures. More importantly though, for the first time it is possible to examine these structures in their natural position in 3D, as well as their configuration with respect to each other. This is a significant advantage compared to traditional histological dissections that always remove all parts from the head and then separate each structure for separate examination. Our 3D volume reconstruction allows detailed examinations from all possible angles and the segmented mouthparts can be observed independently or in combination with each other.

Nevertheless, there are some problems with our 3D reconstructed model. The most problematic structure is the glossa. As already pointed out by Gotwald (1969), due to its highly membranous nature it is deformed in most dead specimens independently of preservation agent. In our specimen, the glossa was already collapsed to a crater-like appearance prior to micro-CT scanning, thus not available for any shape examination.

Another important limitation is that not all structures could be satisfactorily outlined during the segmentation process. This was especially difficult for the delineation of some components, such as the cardo, the lacinial comb, the regions where labium and maxillae meet, and generally everywhere where membranous and chitinous tissues are in contact. These problems are caused by scanning a dry mounted specimen, in which most internal structures have undergone desiccation, shrinkage, and deformation. In such specimens, the dissimilarities in density and contrast between different tissues or components are minimal to zero, thus causing significant problems for the proper recognition and subsequent outlining of borders between structures. In general, our 3D reconstructed model provides fewer details compared to histological dissections. However, these problems might be solvable in future studies if specimens are preserved and prepared in a way more suitable for micro-CT scanning and virtual reconstruction. Based on unpublished data, the use of freshly killed material or specimens in alcohol combined with the use of potassium hydroxide (KOH) and iodine staining provides much better resolution of internal structures than the use of dry material. This allows a much more sophisticated recovery of mouthparts morphology.

The application of micro-CT scanning to obtain information about cuticle thickness is novel. Based on our data however, we refrain from using it for taxonomic diagnostics at the moment. There are some differences in the cuticle thickness among the three species, most notably the very thick head of *Z. obamai* (CCCI 44 vs. CCCI 31 in *Z. sarowiwai* and *Z. wilsoni*). It also appears that the head of *Z. obamai* is thicker than the pronotum and abdominal segments II and III, whereas the heads of the other two species are thinner than or as thin as most other body parts (see Table 5). However, these results could be based on measuring the cuticle at a wrong angle resulting in a distorted result. Even though we have tried to find the best possible sagittal section slides for virtual measuring, it cannot be ruled out that we did not measure the thickest part of the cuticle. For future use, we recommend measuring more slides and more body parts in order to achieve a more complete picture of cuticle thickness. Even though we do not use it for taxonomic diagnostics at the moment, we believe there is potential for such use (unpublished data). However, this should be investigated with more taxa and more specimens first. Nevertheless, our results permit to place the treated species in an evolutionary context and make interpretations about natural history. Currently, the use of micro-CT data for virtual cuticle measuring has rather limited applicability due to the sparse availability of scanning resources in the myrmecological community. Nevertheless, we believe such data provides important information that might be valuable for future systematic and evolutionary studies.

### Cybertypes

As pointed out in previous studies, a crucial advantage of using 3D models based on micro-CT data is its potential application as cybertypes (Faulwetter et al. 2013, 2014; Akkari et al. 2015; Hita Garcia et al. 2017a, 2017b). The concept of a cybertype is to present a detailed and as complete as possible virtual reconstruction of a physical type specimen that is freely accessible. Hita Garcia et al. (2017a) critically assessed the usefulness of cybertypes for ant taxonomy, and due to the limitations in voxel resolution in their scan data suggested to use a combination of micro-CT data (raw data, 3D PDF, and 3D rotation video) and montage photos (three standard view: head in full-face view, full body in profile and dorsal view) as a minimal cybertype for ants. Although we have achieved much higher quality 3D reconstructions compared to previous ant studies using micro-CT and strongly reduced the limitations discussed by Hita Garcia et al. (2017a), we still believe that a minimum ant cybertype should include optical montage photographs. The main reason for this is the lack of natural colour in the micro-CT, which can only be shown with visible light photography. However, in this study we improve the previous ant cybertypes by providing micro-CT scans of single body parts and scan data for the holotype and one paratype, if available, thus increasing the usefulness of the cybertype datasets.

### Virtual character evaluation and presentation

One aim of this study was to evaluate new taxonomic characters for species level taxonomy on the basis of traditional morphological analysis and virtual examination of 3D reconstructions. Unfortunately, only dry mounted material was available for this study. As pointed out in Hita Garcia et al. (2017a), most internal anatomical structures of such specimens have undergone significant desiccation, shrinking and deformation. Consequently, micro-CT data from dry specimens provides much less useful information for comparative examination. Based on our initial investigation, however, some internal sclerotized structures, such as the tentorium, several apodemes, and the endosternum appear to have significant potential for comparative morphology among species. However, due to the poor recovery of these structures in our raw data, we could not examine these in detail and focused our character evaluation on external morphology, with the exception of cuticle thickness. For future taxonomic studies using micro-CT, we propose to examine internal characters in more detail by using material preserved in ethanol.

Initially, our intention was to omit a species identification key, which may appear counterintuitive and substandard. However, there are several problems with dichotomous identification keys that lead us to take a different approach in this study. Identification keys for well-studied regions, such as Japan and Central Europe, generally work well and are very stable since new species are rarely encountered and nomenclatorial benchmarking is rare. This is certainly not the case for most tropical and subtropical regions because our knowledge of the local and regional diversity is fragmentary to non-existent. One major limitation of keys for such regions is that they usually only work for the known species at the time of publication. Later discoveries of new species render keys less useful and often less reliable for identification purposes. To avoid this, it is necessary to update older keys in additional publications after new species are discovered, as done by Hita Garcia and Fischer (2014) to update Hita Garcia et al. (2010). However, this is comparatively work-intensive and most authors of revisionary taxonomy studies are hesitant to revisit previously “finished” groups. This situation is especially problematic in hyperdiverse genera, for which there is no obvious solution except for continuously revising the taxa until most or “all” species are known and described. However, for genera with small or moderate species richness there might be alternatives to traditional identification keys with a few characters per key couplet.

In the case of *Zasphinctus* it is very likely that future collecting in the Afrotropical region will reveal additional species, even though not too many. This assessment is based on the apparent rarity of these ants and the fact that the region is largely undersampled. Accordingly, any identification key that covers only the three species treated here is doomed to obsolescence with the discovery of additional species, especially if only a few characters are listed per key couplet. Instead of simply providing a short key, we decided to present an illustrated matrix with numerous characters, in which we only present the ones that have proven to be diagnostic. Future users of our identification system can check multiple character illustrations and compare them with their specimens at hand. Nevertheless, despite our concerns with a short key with few diagnostic characters, we understand that some users would still prefer to use a short dichotomous key and we still provide one in this study.

Furthermore, the characters chosen are suited for diverse audiences with different interests and resources. For users with limited microscopy resources or little taxonomic training, we have included many easy-to-examine characters that are visible at lower magnifications, such as the shapes of head, mesosoma, or abdominal segment II in profile (e.g. Figs 4A–F, J–L, 5G–R). Based on that, we have added numerous further characters that are not completely necessary for simple identification purposes of faunistic or ecological studies, but target a more taxonomically oriented audience with better microscopy resources and deeper knowledge of ant morphology. We present many characters that require detailed examinations, such as structures on the posterior or ventral head (Figs 4P–R, 5A–F) and the ventral metasoma (Fig. 6A–C, G–L, P–R). These are intended to provide important comparative data for future systematic studies, and will very likely improve delimitations of any additional species.

As outlined above, compared to the taxonomy of most insect groups, the character sets used in the field of ant worker taxonomy are very often rather limited and rely heavily on setation, sculpture, body size, and colour. These are often problematic since they can be highly variable within species and prone to geographic variability, such as shown for the Neotropical *Tatuidris* Brown & Kempf (Donoso 2012), Malagasy *Tetramorium* Mayr (Hita Garcia and Fisher 2011) and Malagasy *Crematogaster* Lund (Blaimer 2012). Against the background of a resilient taxonomic impediment with continuously declining taxonomic resources and funding (e.g. Wheeler et al. 2004; Ebach et al. 2011), it is imperative to deliver taxonomic works that provide high quality species delimitations at a more accelerated speed that offer a stable taxonomic foundation upon which future discoveries can be based. We believe that the application of 21^st^ century taxonomic tools, such as molecular phylogenetics/phylogenomics and 3D next-generation morphology techniques, can strongly improve ant taxonomy.

For the revision of Afrotropical *Zasphinctus*, we have evaluated every single character that could be of diagnostic importance based on the literature record (Bolton 1990; Keller 2011; Borowiec 2016) through a combination of examination of physical material under the light microscope and virtually reconstructed 3D models of micro-CT scans. The latter is of crucial importance since it provides numerous advantages. The most important turned out to be the use of micro-CT data for virtual character examinations and dissections. As noted above, the available material was too scarce to perform physical dissections or dangerous manipulations of specimens. Fortunately, we were able to examine the virtual specimens from all imaginable angles by rotating the 3D models. In addition, in order to examine characters that were hidden behind other body parts, we virtually removed any obstructing structure. By doing this we were able to observe and reveal characters that are challenging to see in most dry-mounted specimens, thus rarely used for ant taxonomy. In particular, we found that the ventral and posterior head possesses a series of useful diagnostic characters, such as the hypostoma (Fig. 5D–F), the vertex (Fig. 4M–O), the occiput (Fig. 4P–R), and several margins around these structures (Fig. 4M–O, 5A–C). The same applies for the ventral metasoma since we found that abdominal segments II and III in ventral view provided some valuable characters, such as the subpetiolar process (Fig. 5M–O, 6A–C) or the prora (Fig. 6J–L). Physical examination of most of these would require damaging the specimens by removing legs, moving the head, separating abdominal segments, or in order to examine the occiput, detaching the head from the pronotum. By using micro-CT based 3D models, we were able to accomplish this with valuable type material without any damage.

Furthermore, the use of virtually reconstructed 3D models permits a quick and effective use of time and resources. Dissections and manipulations of physical specimens are usually very time-consuming, especially if histology and SEM are involved. By contrast, the application of micro-CT scanning enables highly accelerated examinations of morphology compared to these methods (Faulwetter et al. 2013; Friedrich et al. 2014). The initial data generation with a powerful scanner and subsequent 3D reconstructions can be done quickly with minimal effort, if the necessary scanning and visualisation resources are available (see limitations below). The virtual examination of characters is easy and very straightforward. The 3D models can be manipulated in many ways to observe the targeted morphological structures and once a character is in focus one can generate high-quality images within seconds. It is also easy to make images of structures from different angles in order to find the best one for presentation purposes. In our study, we generated several hundred character snapshots within a few days, thus allowing us to choose the most suitable ones for the illustrations used in this study. Generating so many images by using montage photography, SEM, or histology would require much more time and significantly slow down the speed of the publication process.

As already discussed in previous studies employing micro-CT data (Faulwetter et al. 2014; Carbayo et al. 2016; Hita Garcia et al. 2017a), the most important weakness is the limited access to scanning resources since most universities or museums do not have their own micro-CT scanners. Currently, the acquisition of scanners and access to external scanning facilities require substantial economic resources, and this situation will remain unchanged for some time. Some natural history museums and universities already have or are in the process of establishing scanning resources, but presently only a small minority of the taxonomic community has access to the technology. In addition, generating, post-processing, and handling the usually rather large data requires time and technical skills. Nonetheless, as with all new technologies, it is likely that technological and computational developments will reduce the costs of scanning, increase the availability for the taxonomic community, and simplify data management (Faulwetter et al. 2014; Hita Garcia et al. 2017a).

### Functional morphology and lifestyle

As outlined above, there is no knowledge of the natural history of Afrotropical *Zasphinctus*, except that they might live in leaf litter since most specimens were collected in litter samples. Against the background that they are dorylines and that their Australian congeners are predators of other ants, it is likely that the species treated in this study pursue a similar lifestyle. The examination of the micro-CT data generated during this study allows some inference about the lifestyle of the studied species.

As mentioned above, all three Afrotropical *Zasphinctus* species possess a very thick cuticle. Peeters et al. (2017) found the species with the thickest cuticle in relation to body size to be predominantly large ponerine genera, such as *Diacamma* Mayr, *Odontoponera* Mayr, *Leptogenys* Roger, and *Ectomomyrmex* Mayr. However, the thickest cuticle was observed in the species *Ooceraea biroi* (Forel), which is a doryline in relative close phylogenetic proximity to *Zasphinctus*. Based on that result, it is not that surprising that African *Zasphinctus* display such thick cuticle. Notwithstanding that Peeters et al. (2017) found that the best predictors for thick cuticle were body size (larger ants have thicker cuticle) and phylogeny (poneroid ants have thicker cuticle), the thick cuticle of African *Zasphinctus* is likely related to a predatory lifestyle. Based on observations of other *Zasphinctus* species mentioned above, it is highly probable that African *Zasphinctus* are top predators that feed predominantly on other ants, which is also the case in the clonal raider ant *Ooceraea biroi*, which feeds primarily on ant brood. Furthermore, Peeters et al. (2017) concluded that cuticle thickness was also negatively correlated with larger colony size in more phylogenetically derived ant lineages (formicoid clade). Despite a severe lack of observation and natural history data, it appears that African *Zasphinctus* live in small colonies, which is well in accordance with the findings of Peeters et al. (2017).

In *Zasphinctus*, the relative amount of muscles responsible for moving the abdomen seems to be largely increased compared to other ants from the formicoid clade, e.g. *Pheidole* Westwood & *Terataner* Emery, studied in previous publications (Sarnat et al. 2016; Hita Garcia et al. 2017a). It may be tempting to think that the genus *Zasphinctus* – as compared to the majority of ant lineages which have (evolved) greatly reduced abdominal musculature – has retained a more ‘primitive’ and wasp-like internal morphology in its abdomen. Yet, its species have a morphology that makes them rather special among ants: their relatively long abdomen is serially constricted between individually rotating presclerital plates. This apparent adaptation may have gained *Zasphinctus* additional functionality during predation and defence by increasing overall flexibility of use for its well-developed sting apparatus (Fig. 15) in the apical abdominal segment. It seems that other doryline genera such as for example *Eusphinctus* Emery, *Sphinctomyrmex* Mayr, and to a lesser degree possibly some *Cylindromyrmex* Mayr, and *Leptanilloides* Mann have evolved very similar features independently from *Zasphinctus*. It may be interesting to investigate the evolution of these specialised morphologies with respect to the different army ant lifestyles. Since studies on internal ant morphology are generally rare, we have little opportunity to compare our present results with those of others. Hashimoto (1996) for example found that, in some ant subfamilies, the muscles in abdominal segment II and III (petiole and postpetiole) show ‘positional and functional modifications’. Although he also gives an anatomically based discussion on the functional morphology of these modifications, there is no information given on related behavioural or ecological functions nor on musculature modifications in the posterior abdominal segments. With our main focus on the taxonomy of the three newly described species we refrain from further speculations and more detailed analyses in this publication and defer to future internal functional ant morphology studies using micro-CT scanning technology.

## Conclusions

Our study highlights the potential of in-depth comparative morphology analyses for taxonomy founded on a combined investigation of physical specimens under light microscopy and virtual 3D models generated from micro-CT data. Our approach reveals a wealth of morphological characters with high diagnostic potential that we use to successfully delimit species within Afrotropical *Zasphinctus*. Even though the worker caste of ants is highly simplified and the presence of cryptic species in many ant genera is increasingly recognised (e.g. Schlick-Steiner et al. 2006; Seifert 2009), we believe that in many cases the whole range of comparative morphology for alpha taxonomy has not been fully explored yet (Keller 2011). Virtual and interactive examination of morphology and anatomy in 3D can fill the gap and improve our understanding of functionality and homology of characters and provide the means for the discovery of new diagnostic characters (Zimmermann et al. 2011; Blanke and Wesener 2014).

Furthermore, considering the lack of material and apparent rarity of Afrotropical *Zasphinctus*, our study also emphasises the strength of micro-CT scanning as a tool for the non-destructive virtual examination of valuable and scarce type material. Based on our results, micro-CT scanning opens up promising possibilities for the integration of very rare type (and non-type) material into systematic studies, as demonstrated here with the singleton holotype of *Z. wilsoni*.

In general, even though it often appears as if the modern era of molecular systematics has dwarfed the importance of morphology-based systematics, we strongly concur with previous authors that by embracing and employing new technologies, such as micro-CT scanning, the study of morphology can still have a significant impact and remain a strong field in systematic and evolutionary biology (Giribet 2010; Keller 2011; Friedrich et al. 2014). Perhaps the most interesting aspect of using micro-CT for ant taxonomy, however, is the potential to bridge different fields of research. Our study and previous ones for spiny *Pheidole* (Sarnat et al. 2016; Sarnat et al. 2017) show that by examining morphology and anatomy in detail insights about potential behavioural adaptations can be gained. By including new and internal morphological characters in our taxonomic studies we can draw conclusions about and make a connection with functional morphology and ecology. In more holistic approaches combined with statistical analyses and controlled for phylogenetic relationships we can study the evolution of morphological adaptations and learn about the mechanisms that make ants so successful in their respective environments.

## Supplementary Material

XML Treatment for Zasphinctus obamai

XML Treatment for Zasphinctus sarowiwai

XML Treatment for Zasphinctus wilsoni

## Supplementary materials

Supplementary material 1 3D PDF 1 Data type: Adobe PDF file Explanation note: *Zasphinctus obamai* sp. n. holotype worker (CASENT0764125). 3D PDF of volumetric surface rendering model of full body. This dataset is made available under the Open Database License (http://opendatacommons.org/licenses/odbl/1.0/). The Open Database License (ODbL) is a license agreement intended to allow users to freely share, modify, and use this Dataset while maintaining this same freedom for others, provided that the original source and author(s) are credited.Francisco Hita Garcia, Georg Fischer, Cong Liu, Tracy L. Audisio, Evan P. Economo

Supplementary material 2 3D PDF 2 Data type: Adobe PDF file Explanation note: *Zasphinctus obamai* sp. n. paratype worker (CASENT0764127). 3D PDF of volumetric surface rendering model of head (most of antennae virtually removed). This dataset is made available under the Open Database License (http://opendatacommons.org/licenses/odbl/1.0/). The Open Database License (ODbL) is a license agreement intended to allow users to freely share, modify, and use this Dataset while maintaining this same freedom for others, provided that the original source and author(s) are credited.Francisco Hita Garcia, Georg Fischer, Cong Liu, Tracy L. Audisio, Evan P. Economo

Supplementary material 3 3D PDF 3 Data type: Adobe PDF file Explanation note: *Zasphinctus obamai* sp. n. paratype worker (CASENT0764127). 3D PDF of volumetric surface rendering model of mesosoma (head and metasoma mostly virtually removed). This dataset is made available under the Open Database License (http://opendatacommons.org/licenses/odbl/1.0/). The Open Database License (ODbL) is a license agreement intended to allow users to freely share, modify, and use this Dataset while maintaining this same freedom for others, provided that the original source and author(s) are credited.Francisco Hita Garcia, Georg Fischer, Cong Liu, Tracy L. Audisio, Evan P. Economo

Supplementary material 4 3D PDF 4 Data type: Adobe PDF file Explanation note: *Zasphinctus obamai* sp. n. paratype worker (CASENT0764127). 3D PDF of volumetric surface rendering model of metasoma (virtually separated from mesosoma and most of legs virtually removed). This dataset is made available under the Open Database License (http://opendatacommons.org/licenses/odbl/1.0/). The Open Database License (ODbL) is a license agreement intended to allow users to freely share, modify, and use this Dataset while maintaining this same freedom for others, provided that the original source and author(s) are credited.Francisco Hita Garcia, Georg Fischer, Cong Liu, Tracy L. Audisio, Evan P. Economo

Supplementary material 5 3D PDF 5 Data type: Adobe PDF file Explanation note: *Zasphinctus sarowiwai* sp. n. holotype worker (CASENT0764654). 3D PDF of volumetric surface rendering model of full body. This dataset is made available under the Open Database License (http://opendatacommons.org/licenses/odbl/1.0/). The Open Database License (ODbL) is a license agreement intended to allow users to freely share, modify, and use this Dataset while maintaining this same freedom for others, provided that the original source and author(s) are credited.Francisco Hita Garcia, Georg Fischer, Cong Liu, Tracy L. Audisio, Evan P. Economo

Supplementary material 6 3D PDF 6 Data type: Adobe PDF file Explanation note: *Zasphinctus sarowiwai* sp. n. holotype worker (CASENT0764654). 3D PDF of volumetric surface rendering model of head (most of antennae virtually removed). This dataset is made available under the Open Database License (http://opendatacommons.org/licenses/odbl/1.0/). The Open Database License (ODbL) is a license agreement intended to allow users to freely share, modify, and use this Dataset while maintaining this same freedom for others, provided that the original source and author(s) are credited.Francisco Hita Garcia, Georg Fischer, Cong Liu, Tracy L. Audisio, Evan P. Economo

Supplementary material 7 3D PDF 7 Data type: Adobe PDF file Explanation note: *Zasphinctus sarowiwai* sp. n. holotype worker (CASENT0764654). 3D PDF of volumetric surface rendering model of mesosoma (head and metasoma mostly virtually removed). This dataset is made available under the Open Database License (http://opendatacommons.org/licenses/odbl/1.0/). The Open Database License (ODbL) is a license agreement intended to allow users to freely share, modify, and use this Dataset while maintaining this same freedom for others, provided that the original source and author(s) are credited.Francisco Hita Garcia, Georg Fischer, Cong Liu, Tracy L. Audisio, Evan P. Economo

Supplementary material 8 3D PDF 8 Data type: Adobe PDF file Explanation note: *Zasphinctus sarowiwai* sp. n. holotype worker (CASENT0764654). 3D PDF of volumetric surface rendering model of metasoma (virtually separated from mesosoma and most of legs virtually removed). This dataset is made available under the Open Database License (http://opendatacommons.org/licenses/odbl/1.0/). The Open Database License (ODbL) is a license agreement intended to allow users to freely share, modify, and use this Dataset while maintaining this same freedom for others, provided that the original source and author(s) are credited.Francisco Hita Garcia, Georg Fischer, Cong Liu, Tracy L. Audisio, Evan P. Economo

Supplementary material 9 3D PDF 9 Data type: Adobe PDF file Explanation note: *Zasphinctus wilsoni* sp. n. holotype worker (MCZ-ENT-00512764). 3D PDF of volumetric surface rendering model of full body. This dataset is made available under the Open Database License (http://opendatacommons.org/licenses/odbl/1.0/). The Open Database License (ODbL) is a license agreement intended to allow users to freely share, modify, and use this Dataset while maintaining this same freedom for others, provided that the original source and author(s) are credited.Francisco Hita Garcia, Georg Fischer, Cong Liu, Tracy L. Audisio, Evan P. Economo

Supplementary material 10 3D PDF 10 Data type: Adobe PDF file Explanation note: *Zasphinctus wilsoni* sp. n. holotype worker (MCZ-ENT-00512764). 3D PDF of volumetric surface rendering model of head (most of antennae virtually removed). This dataset is made available under the Open Database License (http://opendatacommons.org/licenses/odbl/1.0/). The Open Database License (ODbL) is a license agreement intended to allow users to freely share, modify, and use this Dataset while maintaining this same freedom for others, provided that the original source and author(s) are credited.Francisco Hita Garcia, Georg Fischer, Cong Liu, Tracy L. Audisio, Evan P. Economo

Supplementary material 11 3D PDF 11 Data type: Adobe PDF file Explanation note: *Zasphinctus wilsoni* sp. n. holotype worker (MCZ-ENT-00512764). 3D PDF of volumetric surface rendering model of mesosoma (head and metasoma mostly virtually removed). This dataset is made available under the Open Database License (http://opendatacommons.org/licenses/odbl/1.0/). The Open Database License (ODbL) is a license agreement intended to allow users to freely share, modify, and use this Dataset while maintaining this same freedom for others, provided that the original source and author(s) are credited.Francisco Hita Garcia, Georg Fischer, Cong Liu, Tracy L. Audisio, Evan P. Economo

Supplementary material 12 3D PDF 12 Data type: Adobe PDF file Explanation note: *Zasphinctus wilsoni* sp. n. holotype worker (MCZ-ENT-00512764). 3D PDF of volumetric surface rendering model of metasoma (virtually separated from mesosoma and most of legs virtually removed). This dataset is made available under the Open Database License (http://opendatacommons.org/licenses/odbl/1.0/). The Open Database License (ODbL) is a license agreement intended to allow users to freely share, modify, and use this Dataset while maintaining this same freedom for others, provided that the original source and author(s) are credited.Francisco Hita Garcia, Georg Fischer, Cong Liu, Tracy L. Audisio, Evan P. Economo

Supplementary material 13 3D PDF 13 Data type: Adobe PDF file Explanation note: *Zasphinctus sarowiwai* sp. n. worker (CASENT0764652). 3D PDF of volumetric surface rendering of segmented mouthparts excluding mandibles (green= maxillae; yellow=labrum; orang=labium). This dataset is made available under the Open Database License (http://opendatacommons.org/licenses/odbl/1.0/). The Open Database License (ODbL) is a license agreement intended to allow users to freely share, modify, and use this Dataset while maintaining this same freedom for others, provided that the original source and author(s) are credited.Francisco Hita Garcia, Georg Fischer, Cong Liu, Tracy L. Audisio, Evan P. Economo

Supplementary material 14 Video 1 Data type: Video File (.mov) Explanation note: *Zasphinctus obamai* sp. n. paratype worker (CASENT0764127). 3D rotation video of volumetric surface rendering of head (most of antennae virtually removed). This dataset is made available under the Open Database License (http://opendatacommons.org/licenses/odbl/1.0/). The Open Database License (ODbL) is a license agreement intended to allow users to freely share, modify, and use this Dataset while maintaining this same freedom for others, provided that the original source and author(s) are credited.Francisco Hita Garcia, Georg Fischer, Cong Liu, Tracy L. Audisio, Evan P. Economo

Supplementary material 15 Video 2 Data type: Video File (.mov) Explanation note: *Zasphinctus obamai* sp. n. paratype worker (CASENT0764127). 3D rotation video of volumetric surface rendering of mesosoma (head and metasoma mostly virtually removed). This dataset is made available under the Open Database License (http://opendatacommons.org/licenses/odbl/1.0/). The Open Database License (ODbL) is a license agreement intended to allow users to freely share, modify, and use this Dataset while maintaining this same freedom for others, provided that the original source and author(s) are credited.Francisco Hita Garcia, Georg Fischer, Cong Liu, Tracy L. Audisio, Evan P. Economo

Supplementary material 16 Video 3 Data type: Video File (.mov) Explanation note: *Zasphinctus obamai* sp. n. paratype worker (CASENT0764127). 3D rotation video of volumetric surface rendering of metasoma (virtually separated from mesosoma and most of legs virtually removed). This dataset is made available under the Open Database License (http://opendatacommons.org/licenses/odbl/1.0/). The Open Database License (ODbL) is a license agreement intended to allow users to freely share, modify, and use this Dataset while maintaining this same freedom for others, provided that the original source and author(s) are credited.Francisco Hita Garcia, Georg Fischer, Cong Liu, Tracy L. Audisio, Evan P. Economo

Supplementary material 17 Video 4 Data type: Video File (.mov) Explanation note: *Zasphinctus sarowiwai* sp. n. holotype worker (CASENT0764654). 3D rotation video of volumetric surface rendering of head (antennae virtually removed). This dataset is made available under the Open Database License (http://opendatacommons.org/licenses/odbl/1.0/). The Open Database License (ODbL) is a license agreement intended to allow users to freely share, modify, and use this Dataset while maintaining this same freedom for others, provided that the original source and author(s) are credited.Francisco Hita Garcia, Georg Fischer, Cong Liu, Tracy L. Audisio, Evan P. Economo

Supplementary material 18 Video 5 Data type: Video File (.mov) Explanation note: *Zasphinctus sarowiwai* sp. n. holotype worker (CASENT0764654). 3D rotation video of volumetric surface rendering of mesosoma (head and metasoma mostly virtually removed). This dataset is made available under the Open Database License (http://opendatacommons.org/licenses/odbl/1.0/). The Open Database License (ODbL) is a license agreement intended to allow users to freely share, modify, and use this Dataset while maintaining this same freedom for others, provided that the original source and author(s) are credited.Francisco Hita Garcia, Georg Fischer, Cong Liu, Tracy L. Audisio, Evan P. Economo

Supplementary material 19 Video 6 Data type: Video File (.mov) Explanation note: *Zasphinctus sarowiwai* sp. n. holotype worker (CASENT0764654). 3D rotation video of volumetric surface rendering of metasoma (virtually separated from mesosoma and most of legs virtually removed). This dataset is made available under the Open Database License (http://opendatacommons.org/licenses/odbl/1.0/). The Open Database License (ODbL) is a license agreement intended to allow users to freely share, modify, and use this Dataset while maintaining this same freedom for others, provided that the original source and author(s) are credited.Francisco Hita Garcia, Georg Fischer, Cong Liu, Tracy L. Audisio, Evan P. Economo

Supplementary material 20 Video 7 Data type: Video File (.mov) Explanation note: *Zasphinctus sarowiwai* sp. n. paratype worker (CASENT0764650). 3D rotation video of volumetric surface rendering of full body. This dataset is made available under the Open Database License (http://opendatacommons.org/licenses/odbl/1.0/). The Open Database License (ODbL) is a license agreement intended to allow users to freely share, modify, and use this Dataset while maintaining this same freedom for others, provided that the original source and author(s) are credited.Francisco Hita Garcia, Georg Fischer, Cong Liu, Tracy L. Audisio, Evan P. Economo

Supplementary material 21 Video 8 Data type: Video File (.mov) Explanation note: *Zasphinctus wilsoni* sp. n. holotype worker (MCZ-ENT-00512764). 3D rotation video of volumetric surface rendering of head (antennae virtually removed). This dataset is made available under the Open Database License (http://opendatacommons.org/licenses/odbl/1.0/). The Open Database License (ODbL) is a license agreement intended to allow users to freely share, modify, and use this Dataset while maintaining this same freedom for others, provided that the original source and author(s) are credited.Francisco Hita Garcia, Georg Fischer, Cong Liu, Tracy L. Audisio, Evan P. Economo

Supplementary material 22 Video 9 Data type: Video File (.mov) Explanation note: *Zasphinctus wilsoni* sp. n. holotype worker (MCZ-ENT-00512764). 3D rotation video of volumetric surface rendering of mesosoma (head and metasoma mostly virtually removed). This dataset is made available under the Open Database License (http://opendatacommons.org/licenses/odbl/1.0/). The Open Database License (ODbL) is a license agreement intended to allow users to freely share, modify, and use this Dataset while maintaining this same freedom for others, provided that the original source and author(s) are credited.Francisco Hita Garcia, Georg Fischer, Cong Liu, Tracy L. Audisio, Evan P. Economo

Supplementary material 23 Video 10 Link: https://doi.org/10.3897/zookeys.693.13012.suppl23 Data type: Video File (.mov) Explanation note: *Zasphinctus wilsoni* sp. n. holotype worker (MCZ-ENT-00512764). 3D rotation video of volumetric surface rendering of metasoma (virtually separated from mesosoma and most of legs virtually removed). This dataset is made available under the Open Database License (http://opendatacommons.org/licenses/odbl/1.0/). The Open Database License (ODbL) is a license agreement intended to allow users to freely share, modify, and use this Dataset while maintaining this same freedom for others, provided that the original source and author(s) are credited.Francisco Hita Garcia, Georg Fischer, Cong Liu, Tracy L. Audisio, Evan P. Economo
