# Supplementary figures and images for: Next-generation morphological character discovery and evaluation: an X-ray micro-CT enhanced revision of the ant genus Zasphinctus Wheeler (Hymenoptera, Formicidae, Dorylinae) in the Afrotropics
Source: Zookeys. 2017 Aug 23;(693):33–93. doi: 10.3897/zookeys.693.13012 (PMC5777420; doi:10.3897/zookeys.693.13012)

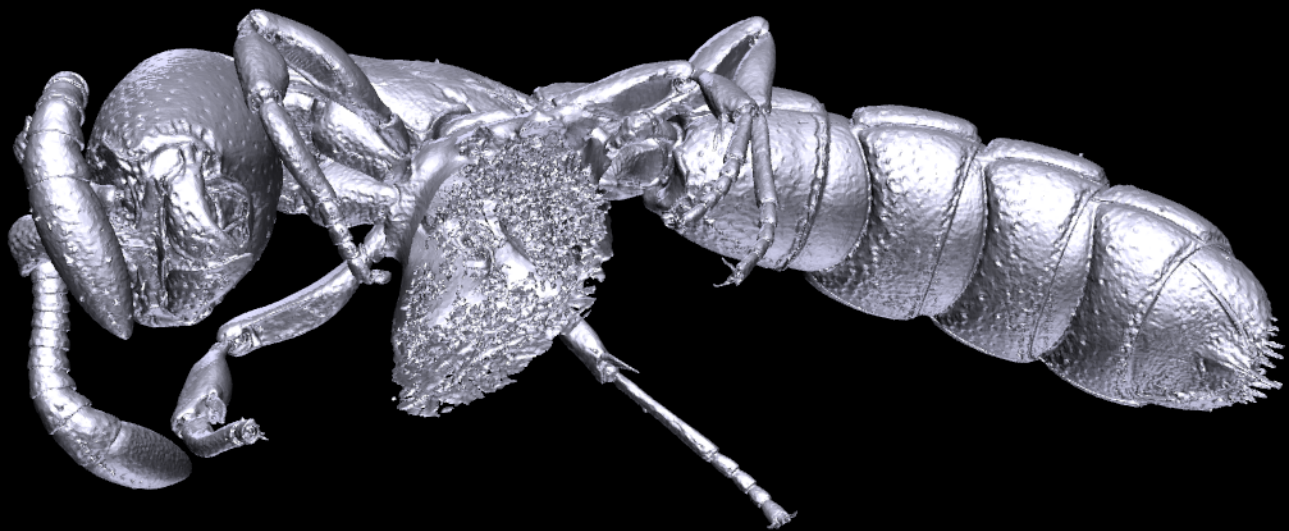

Supplement: Supplementary material 1 — 3D PDF 1 [file zookeys-693-033-s001.pdf]

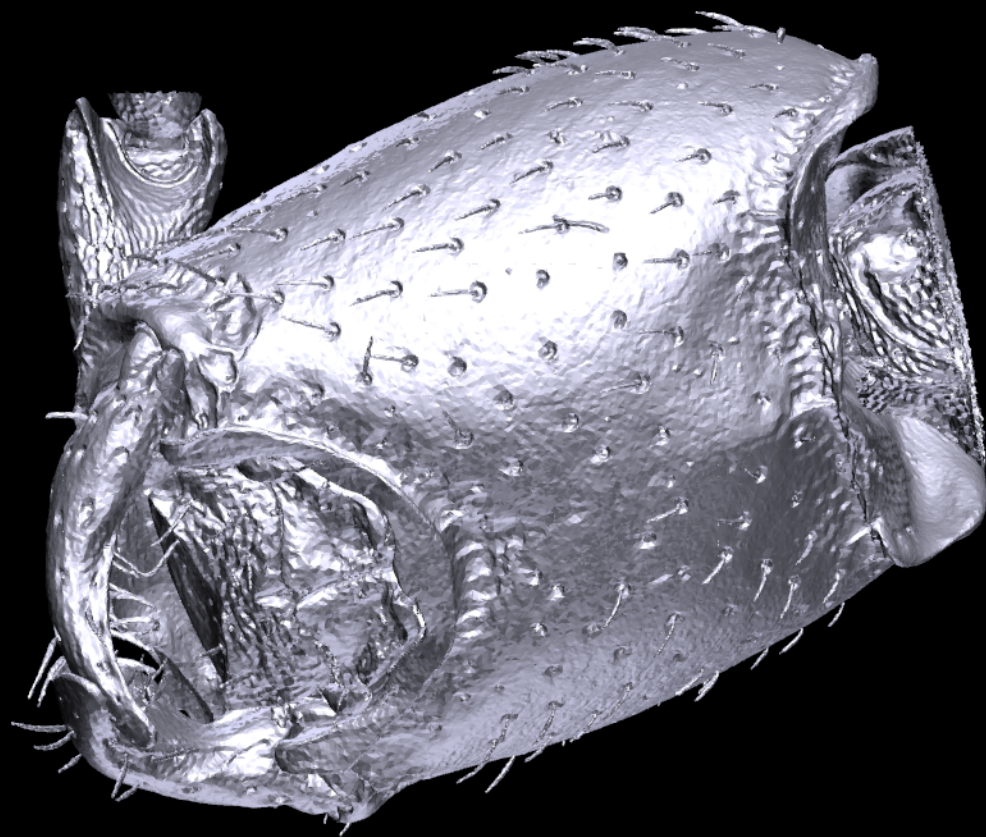

Supplement: Supplementary material 2 — 3D PDF 2 [file zookeys-693-033-s002.pdf]

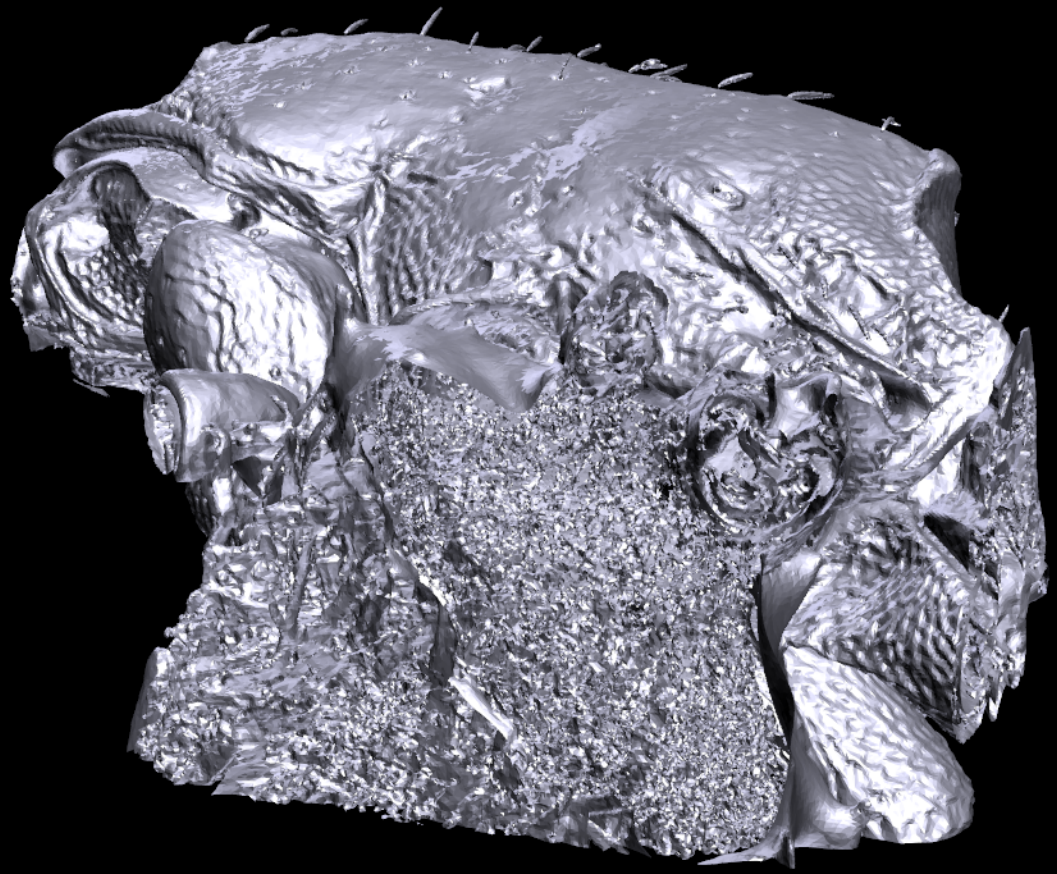

Supplement: Supplementary material 3 — 3D PDF 3 [file zookeys-693-033-s003.pdf]

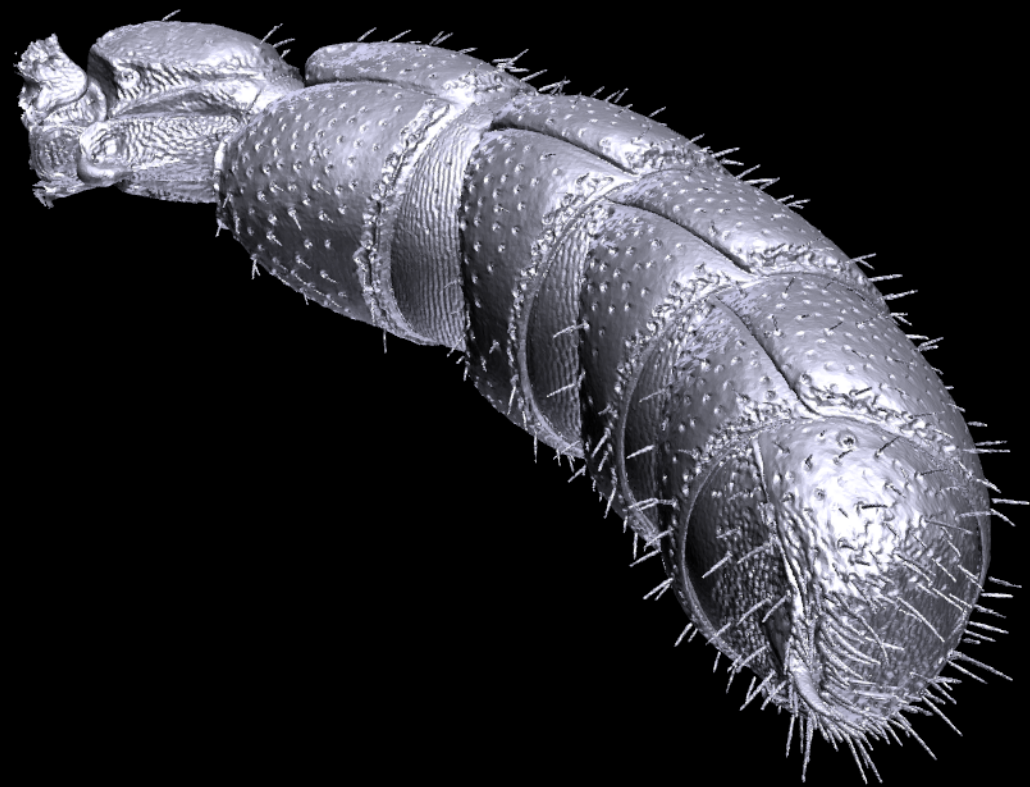

Supplement: Supplementary material 4 — 3D PDF 4 [file zookeys-693-033-s004.pdf]

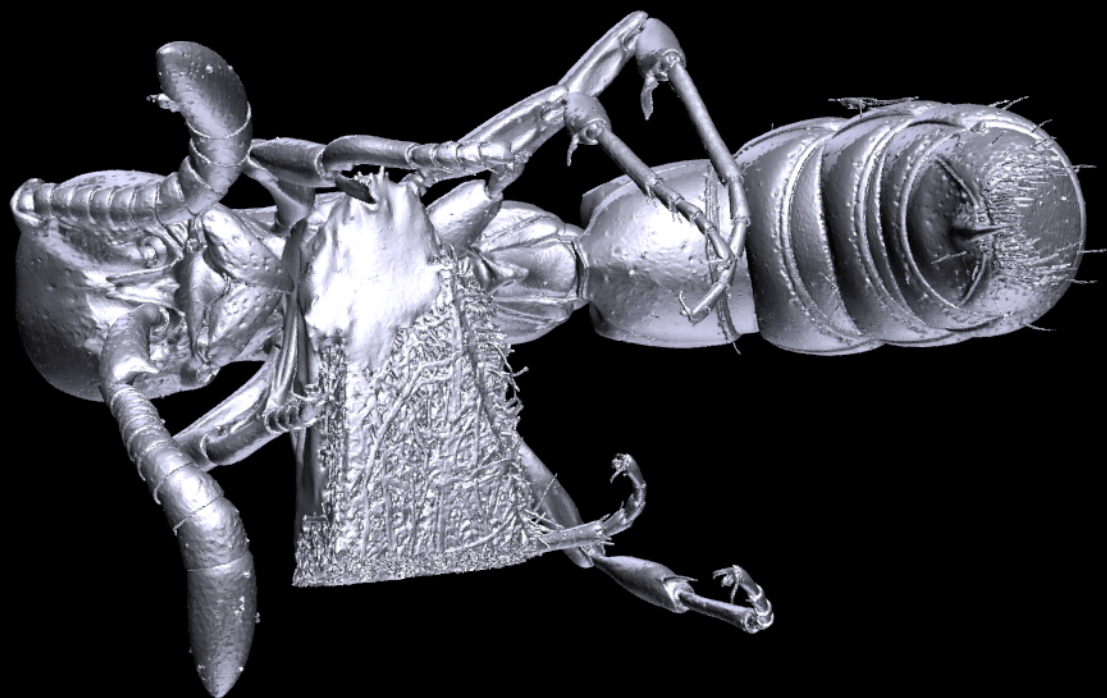

Supplement: Supplementary material 5 — 3D PDF 5 [file zookeys-693-033-s005.pdf]

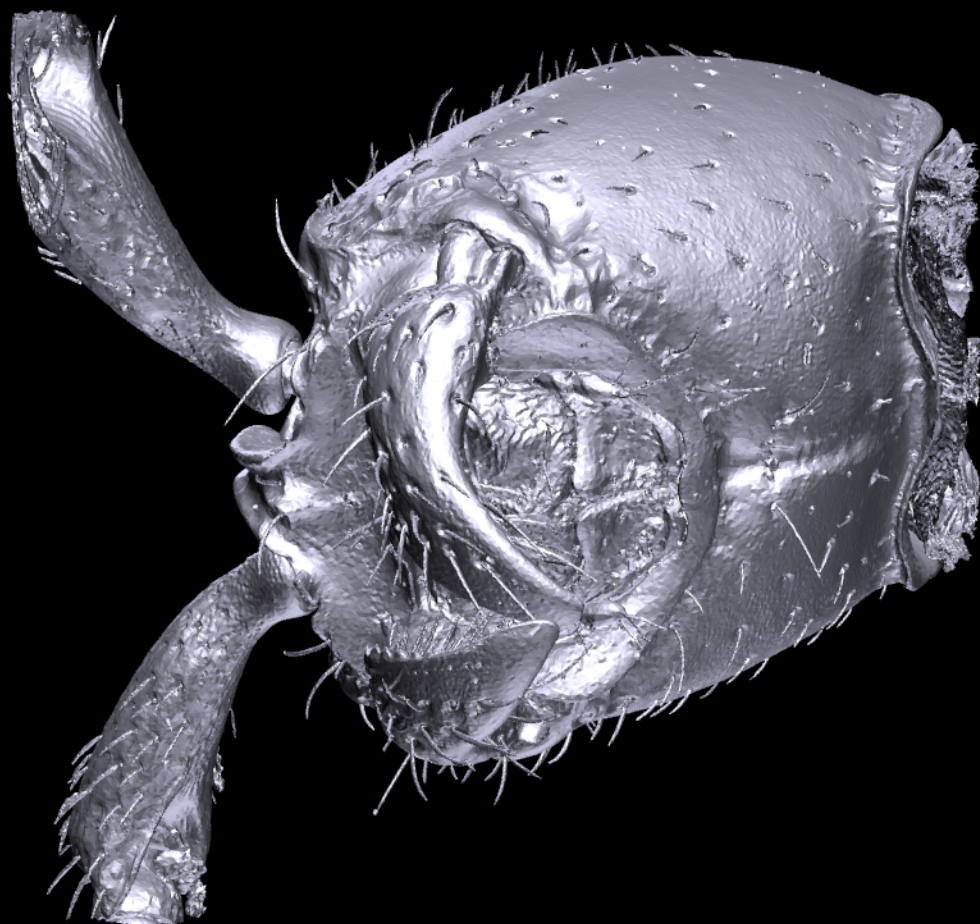

Supplement: Supplementary material 6 — 3D PDF 6 [file zookeys-693-033-s006.pdf]

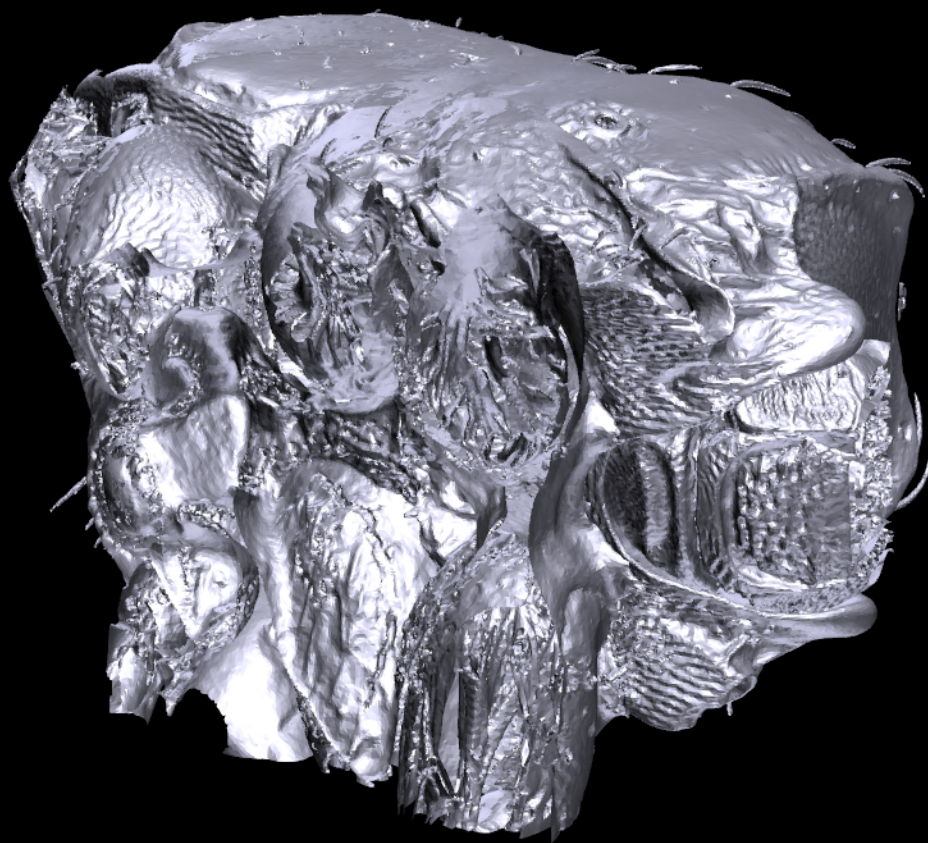

Supplement: Supplementary material 7 — 3D PDF 7 [file zookeys-693-033-s007.pdf]

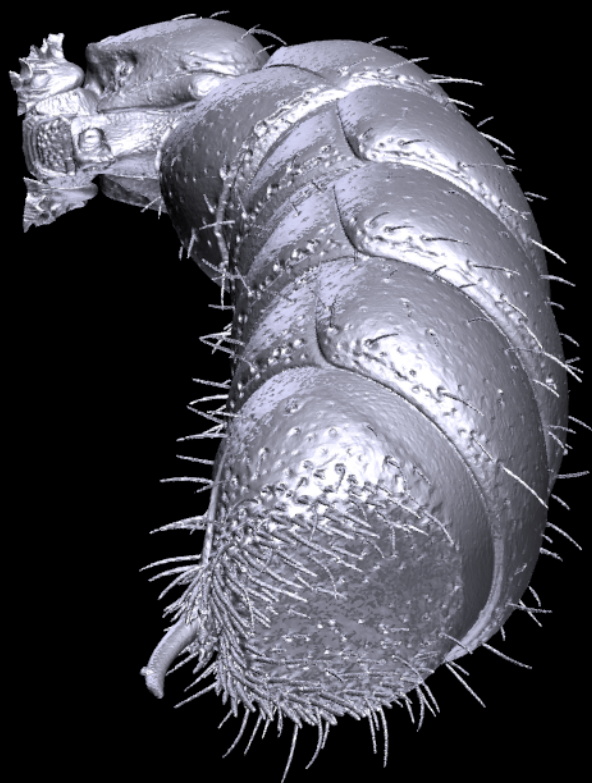

Supplement: Supplementary material 8 — 3D PDF 8 [file zookeys-693-033-s008.pdf]

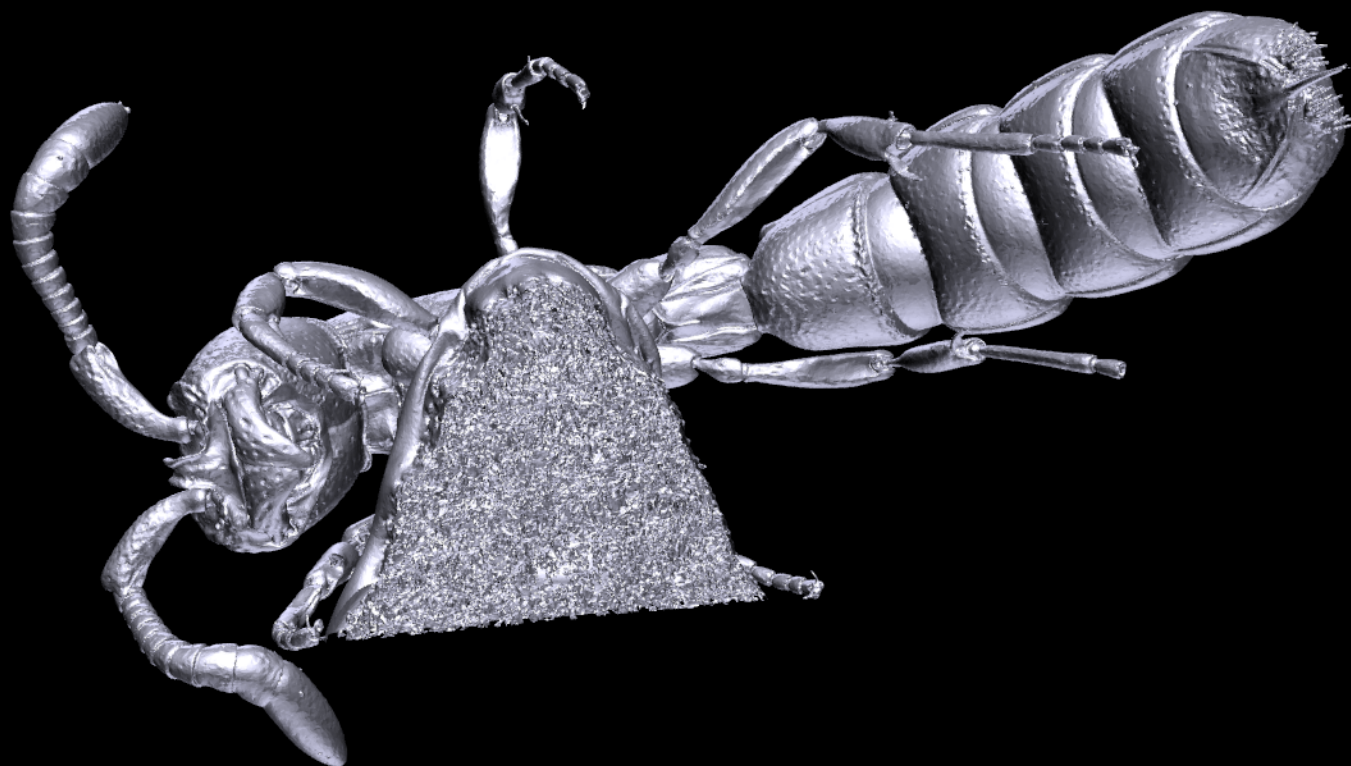

Supplement: Supplementary material 9 — 3D PDF 9 [file zookeys-693-033-s009.pdf]

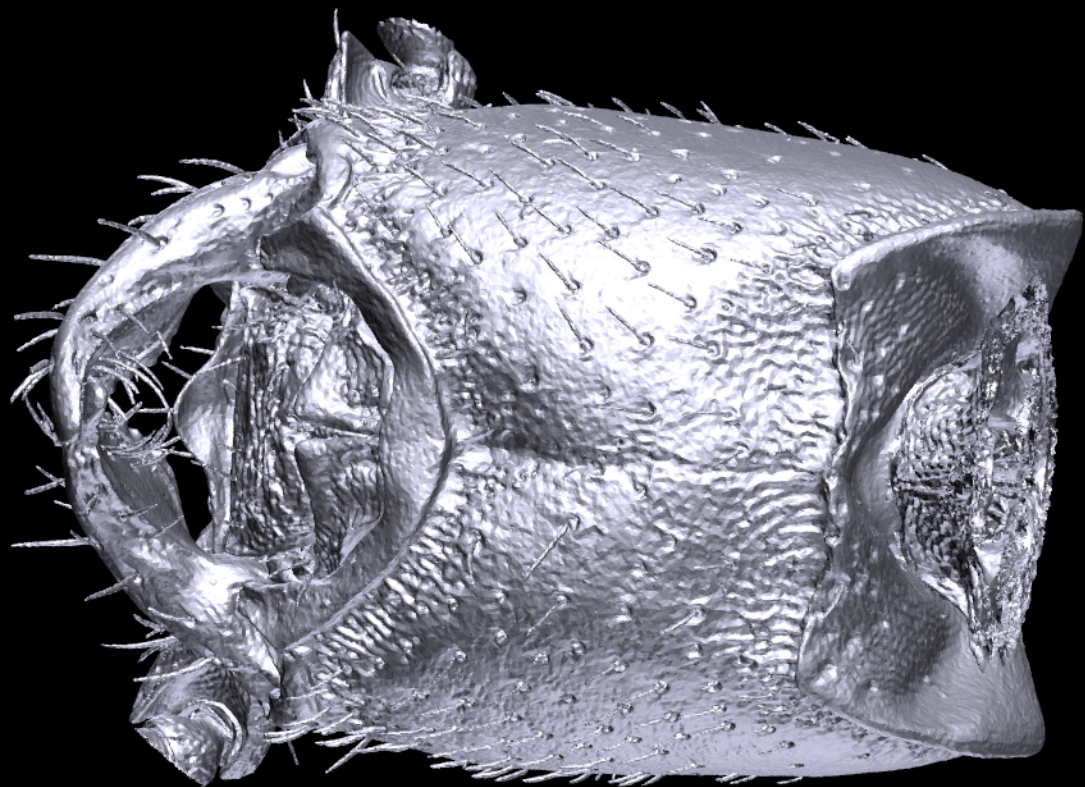

Supplement: Supplementary material 10 — 3D PDF 10 [file zookeys-693-033-s010.pdf]

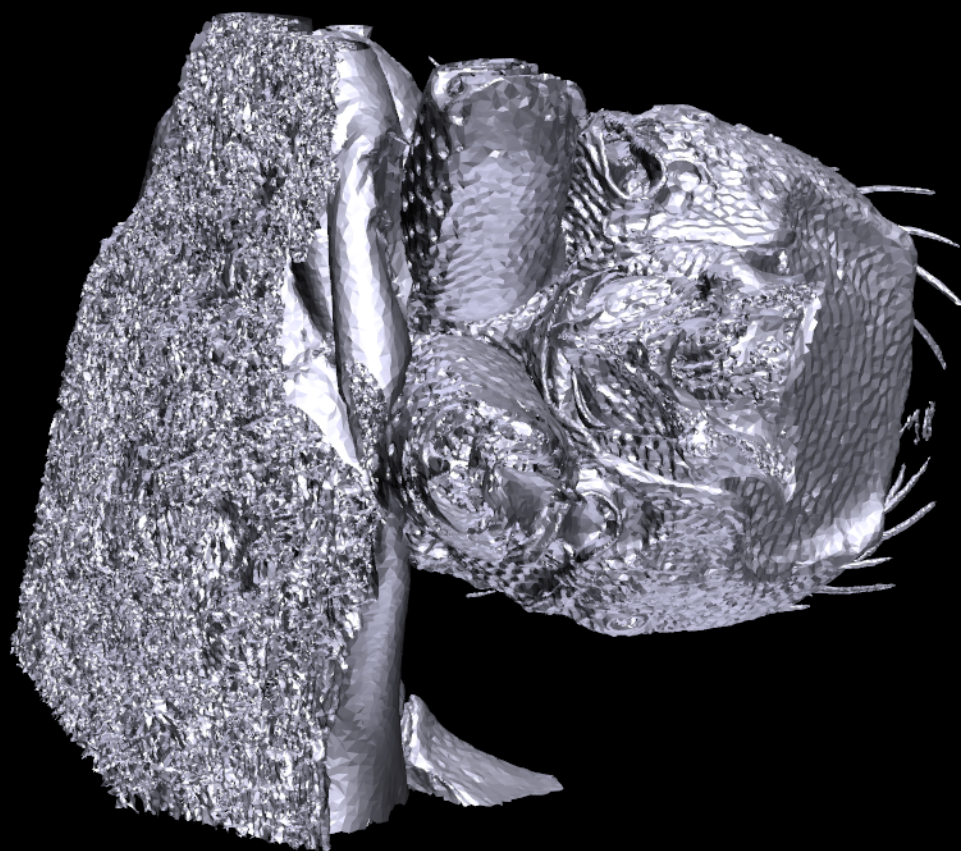

Supplement: Supplementary material 11 — 3D PDF 11 [file zookeys-693-033-s011.pdf]

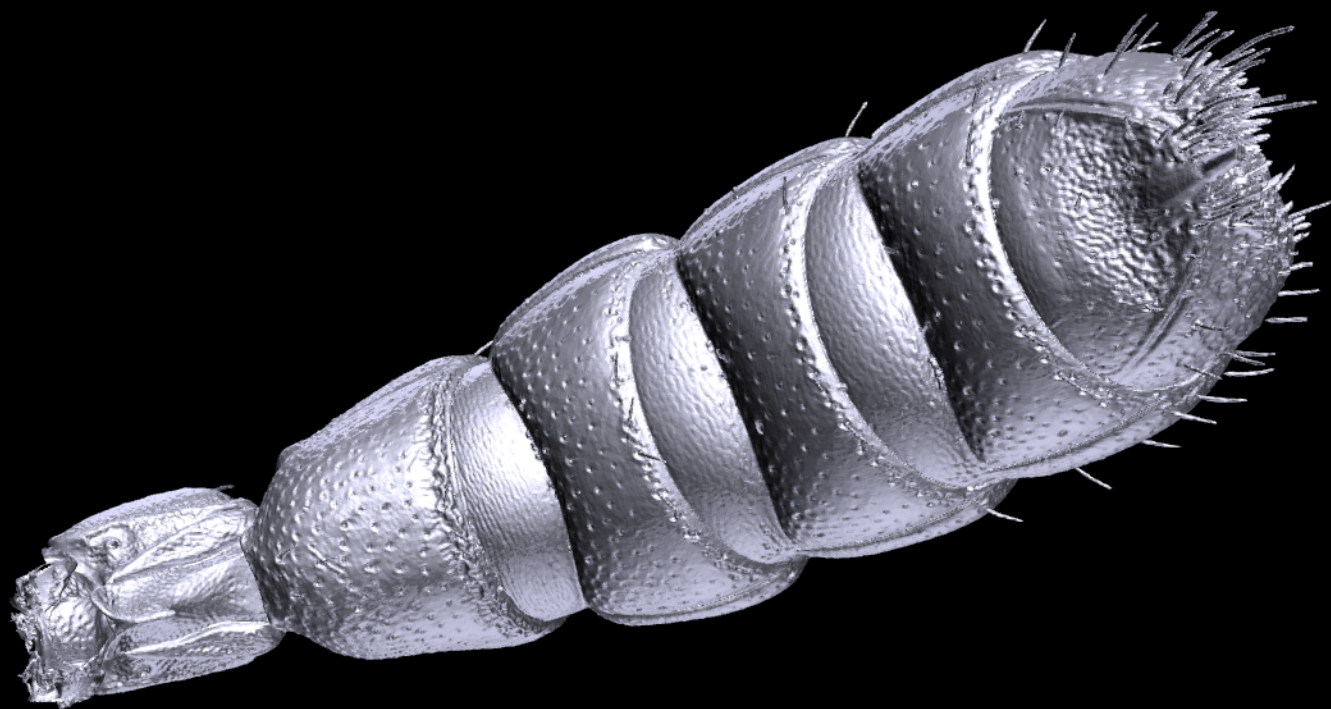

Supplement: Supplementary material 12 — 3D PDF 12 [file zookeys-693-033-s012.pdf]

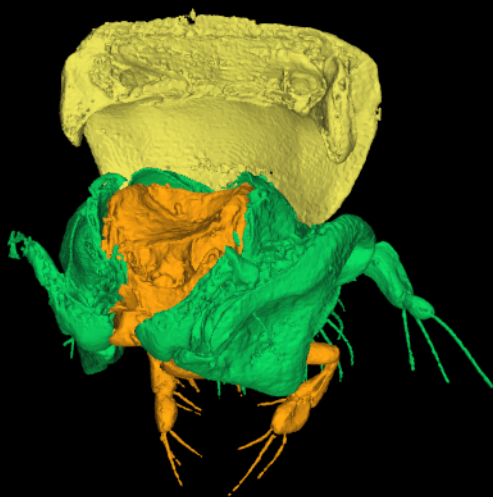

Supplement: Supplementary material 13 — 3D PDF 13 [file zookeys-693-033-s013.pdf]
